# The 8‐oxoguanine DNA glycosylase‐synaptotagmin 7 pathway increases extracellular vesicle release and promotes tumour metastasis during oxidative stress

**DOI:** 10.1002/jev2.12505

**Published:** 2024-09-05

**Authors:** Ying Ma, Jiarong Guo, Haipeng Rao, Jingyu Xin, Xinyi Song, Rui Liu, Shan Shao, Jiajia Hou, Liyu Kong, Zhigang Hu, Lingfeng He, Feiyan Pan, Zhigang Guo

**Affiliations:** ^1^ Jiangsu Key Laboratory for Molecular and Medical Biotechnology, College of Life Sciences Nanjing Normal University Nanjing China

**Keywords:** 8‐oxoG, EVs release, metastasis, OGG1, oxidative stress, SYT7

## Abstract

Reactive oxygen species (ROS)‐induced oxidative DNA damages have been considered the main cause of mutations in genes, which are highly related to carcinogenesis and tumour progression. Extracellular vesicles play an important role in cancer metastasis. However, the precise role of DNA oxidative damage in extracellular vesicles (EVs)‐mediated cancer cell migration and invasion remains unclear. Here, we reveal that ROS‐mediated DNA oxidative damage signalling promotes tumour metastasis through increasing EVs release. Mechanistically, 8‐oxoguanine DNA glycosylase (OGG1) recognises and binds to its substrate 8‐oxo‐7,8‐dihydroguanine (8‐oxoG), recruiting NF‐κB to the synaptotagmin 7 (SYT7) promoter and thereby triggering SYT7 transcription. The upregulation of SYT7 expression leads to increased release of E‐cadherin‐loaded EVs, which depletes intracellular E‐cadherin, thereby inducing epithelial‐mesenchymal transition (EMT). Notably, Th5487, the inhibitor of DNA binding activity of OGG1, blocks the recognition and transmission of oxidative signals, alleviates SYT7 expression and suppresses EVs release, thereby preventing tumour progression in vitro and in vivo. Collectively, our study illuminates the significance of 8‐oxoG/OGG1/SYT7 axis‐driven EVs release in oxidative stress‐induced tumour metastasis. These findings provide a deeper understanding of the molecular basis of cancer progression and offer potential avenues for therapeutic intervention.

## 1 INTRODUCTION

Metastasis, a complex process involving the migration and invasion of primary tumour cells to distant sites, accounts for the majority of cancer‐related mortality (Chambers et al., 2002; Deborde et al., 2022). Developing more effective strategies to combat cancer metastasis requires understanding the detailed mechanisms of metastatic progression. Oxidative stress, defined as an imbalance between reactive oxygen species (ROS) production and the cellular defence system (Sanford et al., 2020; Wang et al., 2022), has been implicated in various pathological conditions, including metastasis (Liou & Storz, 2010; Prasad et al., 2017; Sena & Chandel, 2012; Storz, 2005). Oxidative stress assumes a pivotal role in fostering tumour metastasis through its influence on myriad signalling pathways intricately associated with cancer progression (Hayes et al., 2020; Ishikawa et al., 2008). Foremost among these pathways is the regulation of epithelial‐mesenchymal transition (EMT) – a cellular phenomenon hallmarked by the loss of epithelial characteristics and the acquisition of mesenchymal traits, consequently endowing tumour cells with heightened motility and invasiveness (Fan et al., 2022; Pattabiraman et al., 2016). Oxidative stress elicits EMT induction by activating a panoply of transcription factors, such as SNAIL, Slug and TWIST, which in turn downregulate epithelial markers like E‐cadherin while upregulating mesenchymal markers like N‐cadherin and Vimentin (Chang et al., 2016; Lim et al., 2008; Liu et al., 2012; Son et al., 2023). Furthermore, activation of matrix metalloproteinases (MMPs), a class of proteolytic enzymes responsible for extracellular matrix degradation, is another consequential outcome of oxidative stress (Ho et al., 2011). However, oxidative stress affects not only proteins, but also lipids, nucleic acids and other metabolic molecules. The comprehensive mechanism for the involvement of oxidative stress in metastasis is still largely unknown.

In recent years, extracellular vesicles (EVs) have emerged as pivotal mediators in intercellular communication and have garnered significant attention for their involvement in a range of physiological and pathological processes, notably including the complex phenomenon of tumour metastasis (Jiang et al., 2023; Lee et al., 2023). The small EVs, which include exosomes, have diameters spanning from 30 to 150 nm and are actively released by diverse cell types, including cancer cells (Chen et al., 2022). Exosomes are formed through the endosomal pathway, wherein multivesicular bodies (MVBs) fuse with the plasma membrane, releasing the intraluminal vesicles (ILVs) as exosomes into the extracellular space (Sung et al., 2020). Of paramount importance, tumour‐derived EVs orchestrate a crucial role in the metastatic cascade, facilitating seamless communication and interactions between tumour cells and surrounding microenvironment (Jiang et al., 2020). Laden within these EVs resides a cargo brimming with proteins, lipids, nucleic acids, and other bioactive molecules capable of being transferred to recipient cells, thereby wielding influence over their behaviour and function (Kalluri, 2016). EVs have been demonstrated to augment cancer metastasis through the selective transfer of specific proteins, including MMPs, CD44v6/C1QBP, integrins and epithelial growth factor receptor (EGFR), to the recipient cells. These proteinaceous messengers deftly remodel the extracellular matrix, foster enhanced cellular adhesion, and incite angiogenesis (Hoshino et al., 2015; Shan et al., 2018; Xie et al., 2022; Zhang et al., 2017). Furthermore, EVs excreted by primary tumour cells coordinate the formation of the premetastatic niche (PMN) by establishing a microenvironment that supports metastasis (Jiang et al., 2023; Li et al., 2019, 2024). These EVs contain factors modifying the microenvironment of target organs. Through the transfer of molecules such as oncogenic proteins, integrins, signalling molecules, and miRNAs, they promote angiogenesis, immune suppression, and tissue remodelling, thus effectively nurturing the creation of a propitious terrain conducive to tumour cell colonisation (Wortzel et al., 2019). Therefore, EVs are closely linked to tumour metastasis. Uncovering the details of EVs secretion is critical for understanding cancer progression and treatment. It is already known that EVs release is induced by various physiological conditions such as hypoxia, irradiation and endoplasmic reticulum stress, and is intricately governed by a multitude of genes, encompassing prominent players such as Alix, RAB27A and CD63 (Yu et al., 2020). However, the influence wielded by oxidative stress upon EVs release and the specific regulatory mechanism remain unclear.

Herein, we established a model of low level of oxidative stress‐promoted lung cancer cell metastasis and demonstrated that EVs release was increased in this process. We investigated the underlying mechanism in which the response of 8‐oxoguanine DNA glycosylase (OGG1) to the oxidative signal 8‐oxo‐7,8‐dihydroguanine (8‐oxoG) serves as an upstream event, thereby activating synaptotagmin 7 (SYT7) gene transcription, increasing EVs release and subsequently promoting tumour metastasis. Our data reveal that OGG1/SYT7 axis is important for oxidative stress‐induced tumour metastasis, which could be effectively suppressed by OGG1's DNA binding inhibitor Th5487. Taken together, these findings demonstrate the significance of oxidative signals in the intricate realm of epigenetic regulation and provide a proof of concept for therapeutic targeting of lung metastatic disease.

## 2 MATERIALS AND METHODS

### 2.1 Cell culture and treatment

The human lung adenocarcinoma cell line A549 and human large‐cell lung cancer cell line NCI‐H460 used in this study were purchased from the American Tissue Culture Collection (ATCC) cell bank. The cells were cultured in RPMI‐1640 medium (KGM31800‐500, Keygen Biotech, Nanjing, China) supplemented with 10% foetal bovine serum (FBS) (BC‐SE‐FBS08, BioChannel Biological Technology Co., Ltd., Nanjing, China) at 37°C in a humidified atmosphere with 5% CO_2_. Equivalent numbers of cells were plated into six‐well plate for 12 h, then treated with 20 µM H_2_O_2_ (Shanghai Hushi Laboratorial Equipment Co., Ltd., Shanghai, China) or 10 µM GW4869 (UR21021, Umibio (Shanghai) Co., Ltd., Shanghai, China) for 24 h at 37°C before collection.

### 2.2 RNA interference and generation of knock‐out (KO) stable cell lines

About 3 × 10^5^ A549/NCI‐H460 cells were seeded in six‐well plate for 12 h, and transfected with siRNA duplexes using transfection reagent T101‐01 (Vazyme, Nanjing, China). The final concentration of siRNA was 10 nM and the siRNA sequences were Si‐OGG1‐01: ACACTGGAGTGGTGTACTA, Si‐OGG1‐02: GCAAGTACTTCCAGCTAGA, Si‐OGG1‐03: GGTTCTGCCTTCTGGACAA, Si‐SYT7: GCAAUGACGUCAUCGGCAATT. A random sequence of small interfering RNA fragments (NC‐SiRNA) was used as transfection control.

OGG1 knock out (KO) A549 cells were generated by CRISPR/Cas9 method (target site: KO#1 ACACTGGAGTGGTGTACTAG, KO#2 GGCCTCCAGCTCGTCTGGTG, KO#3 GCTCAACTGTATCACCACTG).

### 2.3 Transwell assay

About 3 × 10^4^ cells were inoculated into the upper chamber of transwell (Corning, NT, USA), and 600 µL culture medium containing 20% FBS was added into the lower chamber. After incubation for 24 h, the cells were fixed with 4% paraformaldehyde (A500684, Sanggong Bioengineering Co., Ltd., Shanghai, China) for 15 min at 37°C and stained with 0.1% crystal violet staining solution (A600331, Sanggong Bioengineering Co., Ltd., Shanghai, China) for 15 min. Three different areas were randomly photographed using an inverted microscope (Nikon, Tokyo, Japan).

### 2.4 RNA extraction and fluorescence real‐time quantitative PCR

Total RNA was extracted from treated cells using FreeZol Reagent (R711‐01, Vazyme Biotech Co., Ltd., Nanjing, China) according to the manufacturer's instructions. For reverse transcription to generate cDNA, the HiScript Q RT SuperMix for qPCR reverse transcription kit (R122‐01, Vazyme, Nanjing, China) was used. The qPCR was performed using AceQ qPCR SYBR Green Master Mix (High ROX Premixed) (Q141‐02, Vazyme, Nanjing, China) with a 20 µL reaction mixture. Each PCR amplification was performed at least three times and repeated in three independent experiments. Primers for OGG1, SYT7 and β‐actin in this study have been listed in Table S1. Analysis of relative gene expression was calculated using the 2^−∆∆Ct^ method. ∆∆Ct = ∆Ct (target gene) − ∆Ct (control, β‐actin). The graphs were plotted using Prism 8.0 (GraphPad Software, San Diego, CA, USA). The differences in mean values between two groups were analysed using Student's *t*‐test.

### 2.5 Western blotting

To lyse and extract the proteins, an appropriate amount of 0.1% SDS lysis buffer (A600485, Sanggong Bioengineering Co., Ltd., Shanghai, China) was added to the treated cells. Approximately 80 µg of each sample was taken for electrophoresis and transferred to PVDF membrane (03010040001, Roche, Switzerland) via eBlot™ L1 rapid wet transfer apparatus (L00686C, GenScript Biotech Corporation, Nanjing, China). After blocking with 5% skim milk powder (P0216‐300 g, Beyotime, Shanghai, China), the PVDF membrane was incubated with primary antibody overnight at 4°C. Then, the membrane was washed three times with PBST buffer, and incubated with secondary antibody for 2 h at room temperature. Finally, ECL chemiluminescence substrate (FD8030, Hangzhou Fude Biotechnology Co., Hangzhou, China) was used for chemiluminescence detection. The membrane was scanned using the Tanon 4500 imaging system (Tanon, Shanghai, China). Grayscale analysis was performed using ImageJ (ImageJ, RRID:SCR_003070). Following grayscale analysis, the relative fold expression of the target gene (target gene/GAPDH) was calculated based on the grayscale value of each band. Graph drawing and statistical analysis were conducted using GraphPad Prism.

The anti‐SYT7 (sc‐293343) and anti‐OGG1 (sc‐376935) antibodies were purchased from Santa Cruz Biotechnology (Dallas, TX, USA) and the anti‐Tubulin (AM1031A) antibody was obtained from Abgent (San Diego, CA, USA). The antibodies against OGG1 (A4997), E‐cadherin (A20798), Vimentin (A19607), CD63 (A5271), Tsg101 (A2216) and Lamin B1 (A16909) were purchased from Abclonal (Wuhan, China). The anti‐*N*‐cadherin (22018‐1‐AP), anti‐CD9 (60232‐1‐Ig), anti‐Alix (67715‐1‐Ig), anti‐Calnexin (66903‐1‐Ig) and anti‐NF‐κB p65 (10745‐1‐AP) antibodies were purchased from Proteintech (Wuhan, China). The anti‐GAPDH (AP0066), goat anti‐mouse IgG (H +L) HRP (BS12478) and goat anti‐rabbit IgG (H +L) HRP (BS13278) antibodies were obtained from Bioworld (Nanjing, China).

### 2.6 Immunofluorescence assay (IF)

The cells were incubated on glass covers overnight, then fixed with 4% paraformaldehyde at room temperature for 10 min and permeabilised with 0.5% Triton X‐100 (IT9100, Solarbio, Beijing, China) for 15 min. Then, they were blocked at room temperature for 2 h in 3% bovine serum albumin (BSA, A8010, Solarbio, Beijing, China). After overnight incubation with primary antibodies at 4°C, glass covers were incubated with corresponding fluorescent secondary antibodies. The nucleus was stained with DAPI (BD5010, Bioworld, Nanjing, China). Image analysis was performed using Nikon confocal inverted microscope (Nikon Instruments, Kawasaki, Japan). Fluorescence intensity was measured using image analysis software (ImageJ). The relative fluorescence intensity multiple (treatment group/NC group) was calculated based on the fluorescence intensity of each image. Statistical analyses and statistical plotting were performed with GraphPad Prism 8.

The anti‐8‐oxoG DNA Lesion (sc‐130914) antibody was purchased from Santa Cruz Biotechnology (Dallas, TX, USA). The antibody against NF‐κB p65 (10745‐1‐AP) was purchased from Proteintech (Wuhan, China). The anti‐phospho‐NF‐κB p65 (#3033S) antibody was purchased from Cell Signalling Technology (Danvers, Massachusetts, USA). The Alexa Fluor ®488 Goat‐Anti‐Rabbit (A‐11008) and Alexa Fluor ®594 Goat‐Anti‐Mouse (A‐11005) were purchased from Life Technologies (Carlsbad, California, USA).

### 2.7 Electrophoretic mobility shift assay (EMSA)

The purified recombinant OGG1 protein (AB98249, Abcam, Cambridge, UK) or extracted nuclear protein was incubated with FAM‐labelled DNA probe (Sangong Biotechnology, Shanghai, China) in a DNA‐binding buffer. Samples were incubated on ice for 10 min and at 37°C for 10 min. Then, the mixture was separated by 7% non‐denatured polyacrylamide gel (40% acrylamide: diacrylamide) in 1 × TBE buffer and imaged by Odyssey FC (Li‐COR Biosciences, Lincoln, NE, United States). The G‐SYT7 probe contained the SYT7 promoter region from −188 to −156 bp. The 8‐oxoG‐SYT7 probe is the corresponding G‐SYT7 sequence containing 8‐oxoG. The cold probe is a probe without FAM label, same as 8‐oxoG‐SYT7. These probes are available in the supplementary materials (Table S1).

### 2.8 Chromatin immunoprecipitation (ChIP) assay

Follow manufacturer's instructions to perform ChIP assays using the kit (P2078, Beyotime, Shanghai, China). The negative control was treated with an equal amount of rabbit (A7016, Beyotime, Shanghai, China) or mouse IgG (A7028, Beyotime, Shanghai, China). Reference IgG was used to verify the specificity of ChIP antibody binding to target genes. Information on primers used in ChIP detection was provided in supplementary materials (Table S1).

### 2.9 Nuclear and cytoplasmic extracts

The nucleoprotein and cytoplasmic protein from A549 cells were extracted according to the nuclear and cytoplasmic extraction kit (CW0199S, CWBIO, Taizhou, China). Briefly, the treated cells were scraped into 1 mL PBS (containing 1 × PMSF) using a cell scrapper. After centrifugation, the cell pellet was collected, and 1 mL Nc‐Buffer A (containing 1 × PMSF) was added to resuspend the pellet. After incubation on ice for 20 min, 55 µL Nc‐Buffer B was added and the samples were incubated on ice for 1 min. After centrifugation for 15 min, the supernatant was collected (this extract was cytoplasmic protein). Then, 500 µL Nc‐Buffer C (containing 1 × PMSF) was added to the pellet obtained in the previous step. Samples were incubated on ice for 40 min and vortexed every 10 min for approximately 15–30 s. Finally, after centrifugation, the supernatant was collected (this extract was nuclear protein).

### 2.10 Streptavidin pull down assay

The nuclear protein from cells treated with 20 µM H_2_O_2_ was extracted. After annealing, the 15 µL (10 µM) double‐stranded biotin‐labelled probe was rotated and incubated with 500 µL A549 cell nuclear extract at 4°C for 12 h in 1 mM PMSF. After washing with PBS, 30 µL streptavidin‐coupled agarose (Pierce, Rockford, IL, USA) was added to precipitate for 2 h. Finally, the DNA–protein complex was washed three times with ice‐cold PBS containing 1 mM PMSF. Western blotting was used to detect the proteins.

### 2.11 MTT assay

For H_2_O_2_ toxicity experiments, 3000 cells were seeded into each well of a 96‐well plate. After treatment with different concentrations of H_2_O_2_ for 24 h, the cells were incubated with MTT solution (50 µg/well, ST316, Beyotime, Shanghai, China) for 4 h, then dissolved with DMSO (196055, MP Biomedicals, LLC, Irvine, CA, USA) and measured at 540 nm (Tecan Infinite F200Pro, Männedorf, Switzerland). For growth curve experiments, the cells were inoculated into 96 well plates at a density of 1000 cells/well and MTT assays were performed daily. All experiments were conducted in three replicates. The relative absorbance value multiple (treatment group/NC group) was calculated based on the absorbance value of each group. The statistical analysis was performed using the GraphPad Prism 8.

### 2.12 ROS detection

An oxidative sensitive fluorescence probe, H2DCFDA (HY‐D0940, MCE, Shanghai, China), was used to detect intracellular ROS. Cells treated with or without 20 µM H_2_O_2_ were incubated with 10 µmol/L H2DCFDA for 20 min at 37°C. Subsequently, after washing cells with PBS, the intracellular ROS production was observed by fluorescence microscope. Fluorescence intensity was measured using image analysis software (ImageJ). The relative fluorescence intensity (fold) (treatment group/NC group) was calculated based on the fluorescence intensity of each image. Statistical analyses and graphing were performed with GraphPad Prism 8.

### 2.13 Colony formation assay

Five hundred cells were seeded in a 6‐well plate. After 1–2 weeks, colonies were counted by crystal violet staining.

### 2.14 Isolation of EVs

About 2 × 10^6^ cells were seeded into a cell culture dish (10 cm). After overnight incubation, the cells were washed three times with PBS and replaced with A549 serum‐free medium (H5B0KJ, Shanghai Basalmedia Technologies, Shanghai, China). The cells were placed in a cell culture incubator at 37°C for 48 h and then the cell supernatant was collected. EVs were collected from cell culture supernatants. The culture supernatant was initially centrifuged at 300 × *g* for 10 min to remove cells, followed by centrifugation at 2000 × *g* for 20 min to remove dead cells and then at 10,000 × *g* for 30 min to remove debris. Subsequently, the supernatant was super centrifuged at 4°C, 100,000 × *g* for 70 min using an Optima XPN‐100 UltraCentrifuge (Beckman, Germany) equipped with a Type 70Ti rotor (Beckman #337922, Brea, CA, USA) and polycarbonate tubes (Beckman #355618, Brea, CA, USA). Acceleration and deceleration settings were both set to 5. After removing the supernatant, the pellet was resuspended in PBS. Following another centrifugation step at 4°C, 100,000 × *g* for 70 min, the pellet was resuspended in 100 µL PBS or SDS lysis buffer (0.1% SDS, 1 × PMSF) for subsequent experiments. EVs samples were stored at −80°C.

### 2.15 Bicinchoninic acid (BCA) assay

The total protein levels in EVs and cells were measured using BCA Protein Assay Kit (CW0014S, CWBIO, Taizhou, China). Briefly, 10 µL cell or EVs lysate was mixed with working solution and incubated for 30 min at 37°C. Absorbance at 570 nm was measured using a microplate reader (Tecan Infinite F200Pro, Männedorf, Switzerland).

### 2.16 Enzyme‐linked immunosorbent assay (ELISA)

The CD63 content in EVs was determined using a CD63 ELISA kit according to the manufacturer's instructions (SBJ‐2507‐96, Sbjbio, Nanjing, China). Briefly, after ultracentrifugation, the EVs were suspended in 100 µL PBS. 40 µL of sample diluent and 10 µL of sample were added to the wells. Wells were incubated at 37°C for 30 min. Next, the washing solution was added into each well and then discarded after stabilising for 30 s, five times repeatedly. After adding 50 µL of enzyme label reagent, the wells were incubated again at 37°C for 30 min. Subsequently, after washing, 50 µL of chromogen A and 50 µL of chromogen B were added to each well. Wells were incubated at 37°C for 15 min. After adding 50 µL of stop solution to each well, the absorbance value of each well was measured at 450 nm using microplate reader (Tecan Infinite F200Pro, Männedorf, Switzerland). Finally, based on the standard curve linear regression equation, each sample concentration was calculated. The relative level of CD63 in EVs (treatment group/NC group) was calculated based on the absorbance value of each sample. The statistical analysis was performed using the GraphPad Prism 8.

### 2.17 Nanoparticle tracking analysis (NTA)

The concentration and size of EVs were measured using ZetaView PMX 110 (Particle Metrix, Meerbusch, Germany), with the corresponding software ZetaView 8.04.02 used for NTA.

### 2.18 Electron microscopy

The purified EVs suspension was added dropwise to the Formvar/carbon‐coated copper grid and stained with 3% phosphotungstic acid aqueous solution for 5 min at room temperature. Then, transmission electron microscope was used to observe the morphology of EVs (H‐7650, Tokyo, Japan).

### 2.19 Zebrafish xenograft experiments

Zebrafish (Suzhou Murui Biotech Co., Ltd., Suzhou, China) were housed in a recirculating aquatic system at 28.5°C with a 12:12 h light/dark cycle and fed dry pellets twice daily according to standard practice. For OGG1‐KO zebrafish xenograft experiments, WT or OGG1‐KO cells were labelled with CM‐Dil fluorescent dye (C7000, Thermo Fisher, MA, USA). The labelled cells were injected into the yolk sac of 2 days postfertilisation (dpf) zebrafish, and zebrafish with relatively consistent fluorescence were selected for experiments. On the third day after injection, zebrafish were photographed. For the maximum tolerated concentration experiment on normal zebrafish, zebrafish juveniles were exposed to different concentrations of Th5487 (HY‐125276, MCE, Shanghai, China) up to 120 h post‐fertilisation (hpf); the solution was changed every 24 h and dead eggs were picked out in time. Statistics were collected on heart rate, mortality, malformation rate and malformation types at 120 hpf. For Th5487 treatment experiments, CM‐Dil labelled A549 cells were injected into the yolk sac of 2 dpf zebrafish, and 20 µM Th5487 treatment was performed 1 day after injection.

### 2.20 Mouse xenograft experiment

In vivo experiments were approved by the Institutional Animal Care and Use Committee (IACUC) of Nanjing Normal University (No. IACUC‐20201202), and the mice were housed in a specific pathogen‐free barrier facility. About 1 × 10^6^ A549 cells were injected into 5‐week‐old mice via tail vein. In the experiment involving OGG1‐KO cells, 1 × 10^6^ fluorescein‐transfected cells were injected into NCG mice (GemPharmatech Co., Ltd, Nanjing, China). After intraperitoneal injection of d‐Luciferin (10 µL/g PBS, ST196, Beyotime, Shanghai, China), bioluminescence images were captured by an IVIS spectrum in vivo imaging system (IVIS Spectrum, PerkinElmer, USA). In the mouse lung metastasis experiment, the metastasis in mouse lung was observed 1 month after cell injection. In the Th5487 treatment experiment, after 10 days of xenograft, BALB/c‐Nude mice (GemPharmatech Co., Ltd., Nanjing, China) received daily intraperitoneal injections of Th5487 (30 mg/kg, 200 µL) daily. Th5487 was dissolved in saline containing 5% DMSO and 10% Tween 80 (IT9000, Solarbio, Beijing, China). After 30 days of Th5487 treatment, the mice were euthanized.

### 2.21 Haematoxylin‐eosin staining (HE)

Lung and liver tissues from NCG or BALB/c‐Nude mice were sent to AiFang Biological (Changsha, China) for paraffin embedding and sectioning. According to the instructions the haematoxylin‐eosin/HE staining kit was used to perform HE staining (G1120, Solarbio, Beijing, China).

### 2.22 Immunohistochemistry (IHC)

Briefly, sections were deparaffinised in xylene and then hydrated in ethanol. Subsequently, 3% H_2_O_2_ solution blocked endogenous peroxidase. Following antigen retrieval with a repair solution, 3% BSA blocking solution was used to block non‐specific binding sites. The anti‐SYT7 and anti‐OGG1 primary antibodies were incubated with sections overnight at 4°C. After washing with PBST, the sections were incubated with the corresponding Polymer‐HRP secondary antibody (AFIHC001, AiFang Biological, Changsha, China) and then developed with DAB (AFIHC001, AiFang Biological, Changsha, China). Finally, the tissue was counterstained with haematoxylin, dehydrated, and mounted.

### 2.23 Dual luciferase reporter gene assay

Luciferase assay was performed in A549 cells co‐transfected with a luciferase reporter plasmid (pGL3‐basic) containing SYT7 promoter region from −348 to −1 bp (synthesised by GenScript Biotech Corporation, Nanjing, China) and a plasmid encoding renilla luciferase (pRL‐TK), following the manufacturer's instructions (RG027, Beyotime, Shanghai, China).

### 2.24 Statistical analysis

Statistical analysis of data was performed using Prism 8.0 (GraphPad Software). The mean or ±SD values ​​calculated from at least three independent experiments were used as statistical data. Student's *t*‐test was used to calculate *P*‐values. *P* < 0.05 was considered statistically significant. The zebrafish juvenile survival rate and heart rate were calculated using conventional one‐way ANOVA test. ns stands for no significance.

## 3 RESULTS

### 3.1 EVs release is critical in oxidative stress‐induced cancer cell migration and invasion

**FIGURE 1:**
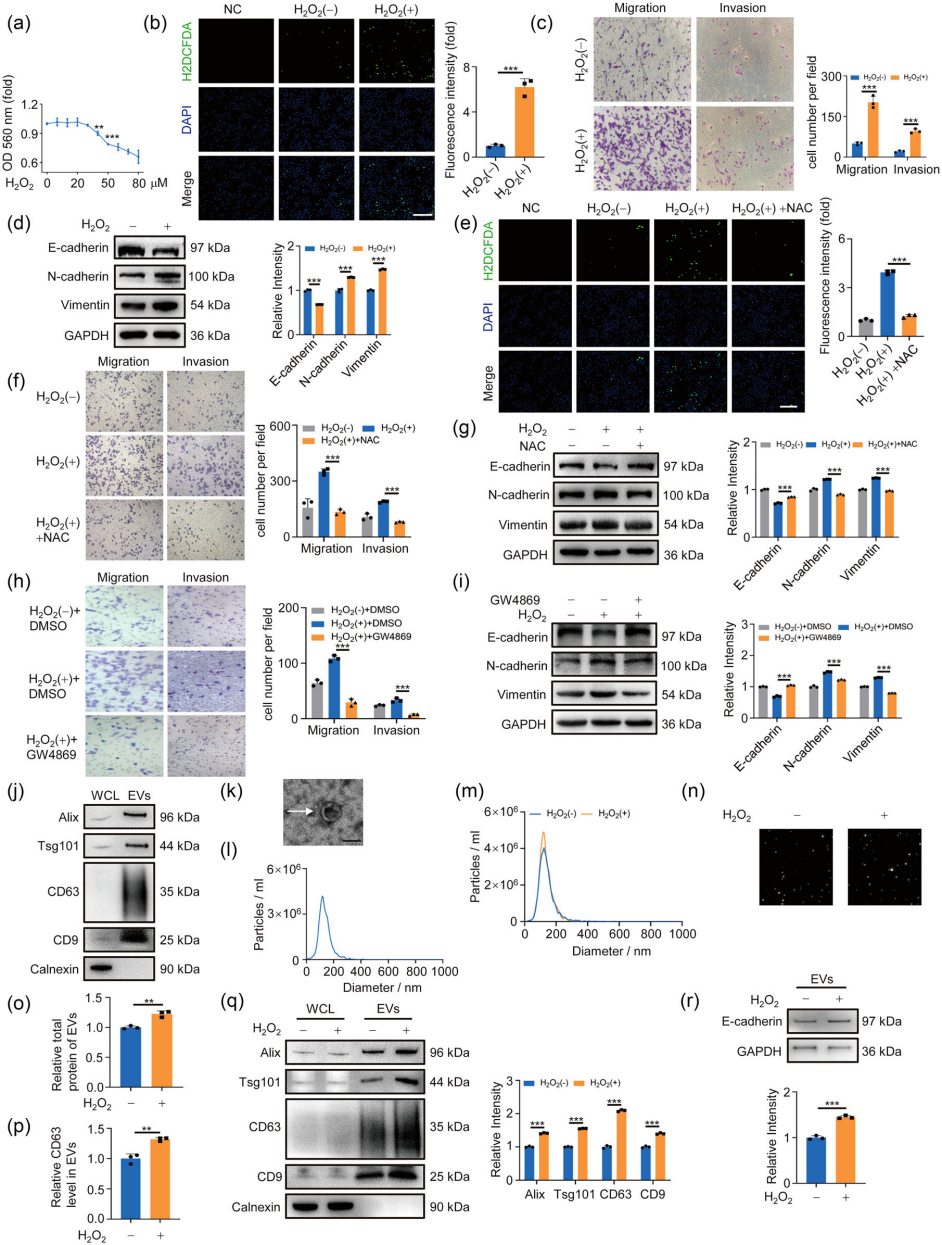
Release of EVs is critical in oxidative stress‐mediated migration and invasion of cancer cells. (A) The effect of H_2_O_2_ concentration gradient on the activity of A549 cells was detected by MTT assay at 24 h. (B) Detection of intracellular ROS generation in A549 cells exposed to 20 µM H_2_O_2_. Scale bar: 200 µm. Right, quantification of corresponding fluorescence intensity. (C) A549 cells were treated with or without 20 µM H_2_O_2_ for 24 h. Crystal violet staining for migrated cells. Right, quantification of migrated cells. (D) Western blotting analysis of EMT markers after 20 µM H_2_O_2_ exposure. Right, quantification of marks. (E) Detection of intracellular ROS in A549 cells exposed to 20 µM H_2_O_2_ and 10 mM NAC. Scale bar: 200 µm. Right, quantification of the corresponding fluorescence intensity. (F) A549 cells treated with 20 µM H_2_O_2_ and 10 mM NAC for 24 h were subjected to transwell assay. Right panel quantified migrated cells. (G) Cells were treated as panel (F) and analysed by Western blotting. Right, quantification of EMT markers. (H) Transwell assay detected the migration of cells exposed to 10 µM GW4869 for 24 h. Right, quantification of migrated cells. (I) Cells were treated as panel (H). Western blotting was used to detect EMT markers expression. Right, quantitative analysis of EMT markers. (J) Western blotting analysis of EVs markers (Alix, Tsg101, CD63 and CD9) and Calnexin as negative marker protein. Right, quantification of marks. (K) EVs were photographed by transmission electron microscopy. Scale bar: 100 nm. (L) Concentration and diameter of isolated EVs were detected by NTA. (M) NTA detected the concentration of EVs after oxidative stress exposure. (N) Screenshot from NTA video. (O) BCA detected the total protein amount in EVs. (P) ELISA detected the content of CD63 in EVs. (Q) Equal amounts of cell‐isolated EVs were analysed for marker protein content by Western blotting. Right, quantitative analysis of markers. (R) Western blotting analysis of E‐cadherin in equal amounts of EVs. GAPDH was used as a loading control. Right, quantification of E‐cadherin. All of the data are expressed as mean values ± SEM (*n* = 3); **p < 0.01, ***p < 0.001. (Student's *t*‐test).

The effect of low‐level oxidative stress on tumour metastasis is an important aspect in unravelling the early stages of tumourigenesis. In this study, we chose the oxidative reagent H_2_O_2_ to treat lung cancer cells. After cell viability assay and intracellular ROS level test for 24 and 48 h, 20 µM was determined as an appropriate concentration to mimic the physiological condition of low‐level oxidative stress (Figures 1A,B and S1A,B). Since NRF2 and p53 are directly related to the occurrence of oxidative stress (Gasparro et al., 2023), the increased protein levels confirmed the induction of oxidative stress (Figure S1C). Next, the transwell assay demonstrated that 20 µM H_2_O_2_ treatment effectively augmented the migration and invasion of lung cancer cells (Figures 1C and S1D). Oxidative stress exposure resulted in the loss of expression of the epithelial adhesion protein E‐cadherin and concomitant increased levels of N‐cadherin and mesenchymal Vimentin, which are crucial markers of EMT (Figures 1D and S1E). The expression of E‐cadherin and N‐cadherin is regulated by transcription factors such as SNAIL and TWIST (Fan et al., 2022; Liu et al., 2017). Oxidative stress increased the protein levels of SNAIL1 and TWIST1 (Figure S1F). Furthermore, this oxidative treatment also increased cell proliferation (Figure S1G,H). To further emphasise the role of oxidative stress in migration and invasion, the free radical scavenger *N*‐acetyl‐l‐cysteine (NAC), an acetylated derivative of cysteine commonly employed as an antioxidant, was applied in the next experiments. Our data showed that NAC exposure led to a reduction in intracellular ROS levels as well as NRF2 and p53 protein levels, and mitigated the augmented migration and invasion induced by oxidative stress (Figures 1E–G, S1I,J), further confirming the positive effect of oxidative stress on cell migration and invasion.

It is well established that tumour‐derived EVs play an important role in the pre‐metastatic microenvironment (Lobb et al., 2017; Qi et al., 2022; Zhang et al., 2017). To investigate whether EVs were involved in oxidative stress‐promoted cell migration and invasion, we employed GW4869, the inhibitor of exosome synthesis/release, to treat the cells. Results showed that 10 µM GW4869 exposure attenuated oxidative stress‐induced cell migration and invasion (Figure 1H,I), indicating the pivotal involvement of EVs in this process. Meanwhile, oxidative stress‐induced cell proliferation was also inhibited by GW4869 treatment (Figure S1K). Next, we isolated EVs from cell culture supernatant by ultracentrifugation and verified their physicochemical properties, such as shape, size and characteristic proteins, by NTA, western blot and electron microscopy according to MISEV guidelines 2018 (Théry et al., 2018) (Figure 1J–L). To delve deeper into the effect of oxidative stress on EVs secretion, we compared EVs released from the same number of cells treated with or without H_2_O_2_ for 48 h (Figure S1L). NTA data and a screenshot of the video recorded during NTA demonstrated a significantly higher EVs count in the oxidative stress group (Figure 1M,N). Furthermore, the total protein amount of the isolated EVs was quantified using the BCA kit and it showed a noticeable increase in the oxidative stress group (Figure 1O). Since EVs were isolated from equal amounts of cells, the abundance of EVs marker proteins reflected the quantity of EVs secreted from cells. Thus, the upregulated expression of marker proteins in EVs further confirmed the positive effect of low‐level oxidative stress on EVs secretion (Figure 1P,Q). Importantly, we observed that oxidative stress exposure for 24 h and 48 h led to an increase in E‐cadherin level within EVs (Figures 1R and S1M), suggesting that a portion of the reduction in intracellular E‐cadherin caused by oxidative stress may be released through the EVs pathway. Altogether, these data suggested that oxidative stress promoted tumour cell migration and invasion partly due to increased EVs release.

### 3.2 OGG1 is involved in tumour metastasis under oxidative stress

**FIGURE 2:**
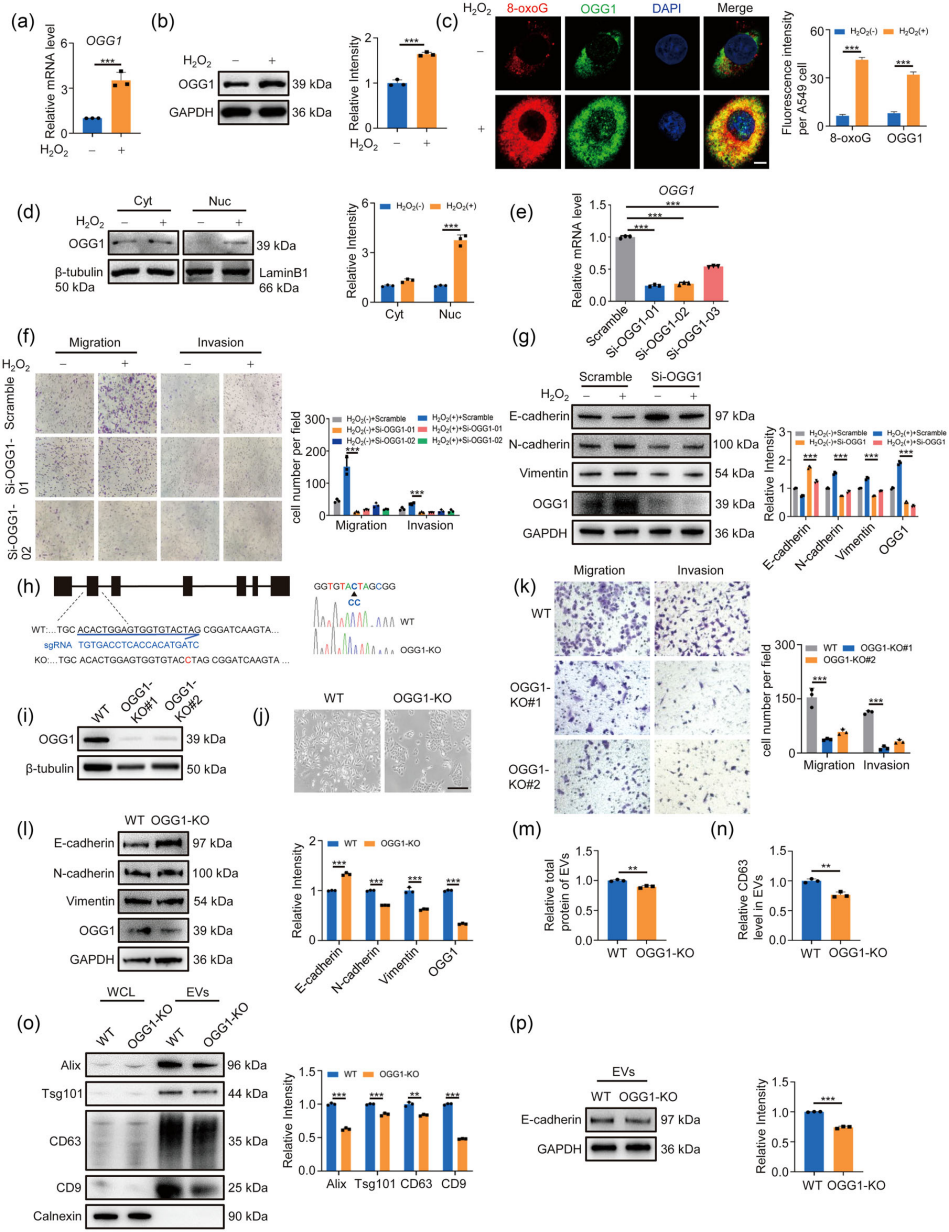
OGG1 knockdown reduces cell migration via EVs release. (A, B) A549 cells were treated with or without 20 µM H_2_O_2_ for 24 h. The mRNA (A) and protein (B) levels of OGG1 were analysed by RT‐qPCR and Western blotting. Right, quantification of OGG1. (C) OGG1 and 8‐oxoG were detected by IF experiment. Colocalisation of 8‐oxoG (red) and OGG1 (green) in cells appeared yellow in the merged images. Scale bar: 5 µm. Right, quantification of immunostaining for 8‐oxoG and OGG1. (D) The cytoplasmic and nuclear fractions were separated and subjected to western blotting to determine the OGG1 level in the cytoplasm and nucleus. Lamin B1 and β‐tubulin were used as internal controls for the nuclear and cytoplasmic fractions. Right, quantification of OGG1 level. (E) RT‐qPCR detection of OGG1 mRNA level in cells transfected with siRNA. (F) OGG1 was knocked down by siRNA in A549 cells. After 24 h, the cells were digested and used for transwell assay. Right, quantification of migrated cells. (G) Western blotting was used to measure EMT markers. Right, quantification of markers. (H) Schematic diagram of CRISPR/Cas9 targeting OGG1. (I) Western blotting was used to detect OGG1 protein level in OGG1‐KO cells. (J) Morphological images of WT and OGG1‐KO cells. Scale bar: 100 µm. (K) Transwell assay was used to detect cell migration and invasion in OGG1‐KO cells. Right, quantification of migrated cells. (L) The level of EMT markers in WT and OGG1‐KO cells was detected by western blotting. Right, quantification of markers. (M) The total protein concentration of isolated EVs was determined using BCA. (N) ELISA determined the content of CD63 in isolated EVs. (O) Western blotting analysed equal amounts of cell‐isolated EVs for markers content. Right, quantitative analysis of markers. (P) The content of E‐cadherin in equal amounts of EVs was quantified by Western blotting. All of the data are expressed as mean values ± SEM (*n* = 3); **p < 0.01, ***p < 0.001. (Student's *t*‐test).

OGG1, as the first responder to oxidative stress, serves as the main protein that repairs oxidative DNA damage (8‐oxoG) caused by ROS (Wang et al., 2018). As expected, the elevated mRNA and protein level of OGG1 were detected after H_2_O_2_ stimulation (Figures 2A,B and S2A,B). Since the intracellular distribution of OGG1 is closely related to its function, we examined the changes in the localisation of OGG1 pre and post exposure to oxidative stress using immunofluorescence staining and nucleocytoplasmic separation analysis. The data indicated that oxidative stress remarkably increased nuclear OGG1 (Figure 2C,D), implying the critical role of nuclear OGG1 in this response. Next, to explore whether OGG1 is involved in lung cancer metastasis under oxidative stress, we first conducted OGG1 loss‐of‐function experiments with small interfering RNA (siRNA). Following successful knockdown of OGG1 (Figure 2E), the oxidative stress‐induced cell migration and invasion were significantly weakened and EMT markers were changed accordingly (Figures 2F,G and S2C–E). Furthermore, we knocked out OGG1 using CRISPR/Cas9 technology (Figure 2H,I) and the results were consistent with those obtained from OGG1‐knockdown cells, highlighting the criticality of OGG1 in mediating oxidative stress‐induced tumour migration and invasion (Figures 2J–L and S2F). Noteworthy, the loss of OGG1 significantly reduced the colony‐forming efficiency (Figure S2G). Given our substantiated evidence regarding the pivotal role played by EVs release in oxidative stress‐induced cell migration and invasion, we next wondered whether OGG1, as the oxidative DNA sensor, could regulate EVs secretion to promote tumour metastasis. Therefore, we examined EVs isolated from cell supernatants after OGG1 knockout (Figure S2H,I). The results demonstrated that OGG1 deletion significantly inhibited EVs secretion and concurrently reduced the E‐cadherin loaded in EVs, as evidenced by BCA of total EVs protein, ELISA of CD63 content, and western blot analysis of EVs markers (Figure 2M–P). Altogether, these results suggested that OGG1 has important functions in EVs release and tumour metastasis when responding to oxidative stress.

### 3.3 *SYT7* is a new target gene of OGG1

**FIGURE 3:**
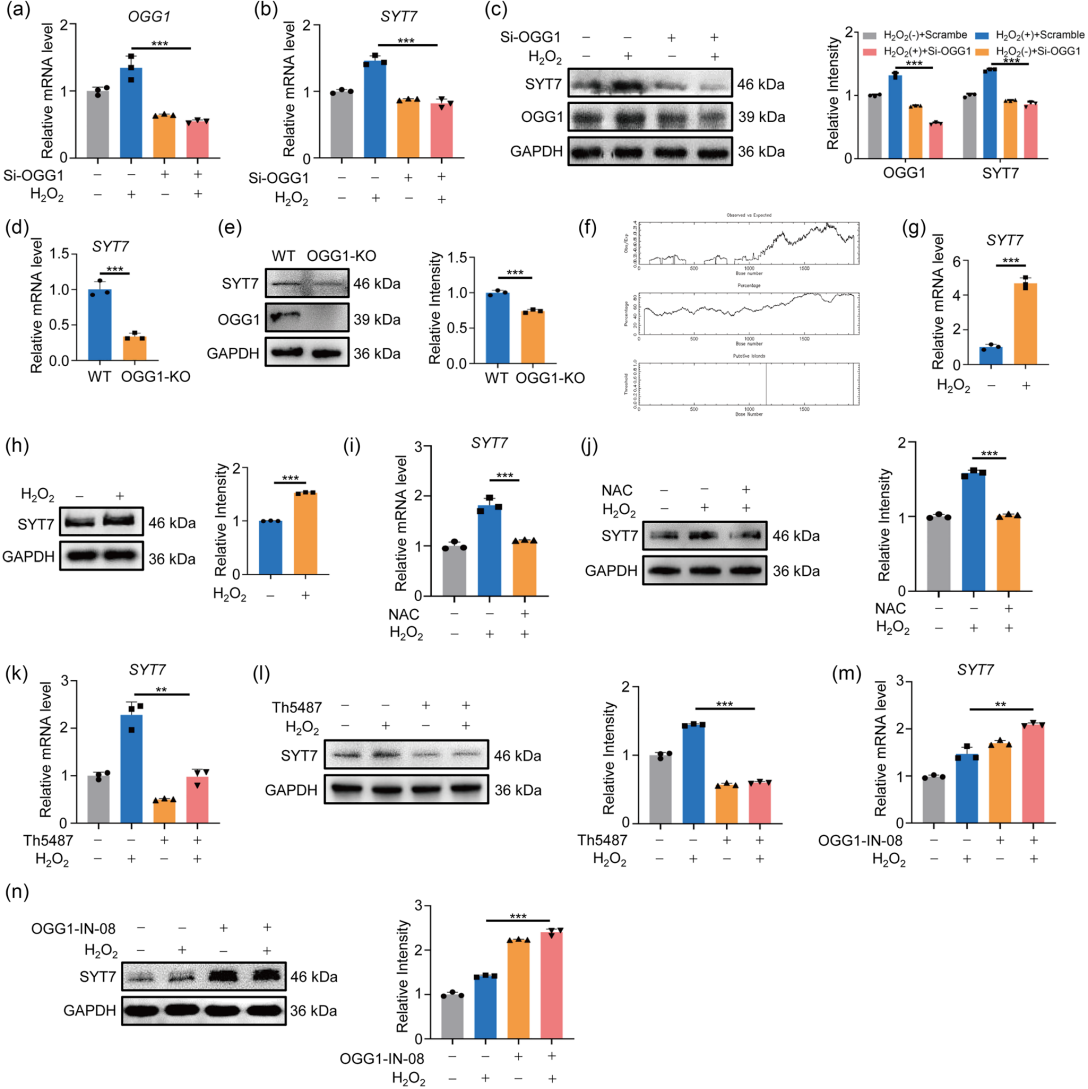
SYT7 is a potential target gene of OGG1. (A, B) After A549 cells were transfected with scrambled or OGG1 siRNA for 48 h, the mRNA levels of OGG1 (A) and SYT7 (B) were examined. (C) Cells were treated as panel (A). Western blotting was used to determine the protein levels of OGG1 and SYT7. Right, quantification of OGG1 and SYT7. (D, E) The mRNA (D) and protein (E) levels of SYT7 in the WT and OGG1‐KO cells were examined by RT‐qPCR and Western blotting. (F) Analysis of CpG islands 2 kb upstream of the TSS region of SYT7. Upper graph: A plot of the ratio of observed GC content to expected GC content. Middle graph: GC content percentage. Lower graph: The CpG island was predicted to exist at −844 to −55 nucleotides (nt) upstream of the TSS region of SYT7. (G, H) The mRNA (G) and protein (H) levels of SYT7 in A549 cells treated with or without 20 µM H_2_O_2_ were analysed. Right, quantification of SYT7 level. (I, J) After cells were treated with 20 µM H_2_O_2_ and 10 mM NAC for 24 h, the mRNA (I) and protein (J) levels of SYT7 were analysed. Right, quantification of SYT7. (K, L) After cells were treated with or without 10 µM Th5487 for 24 h, the mRNA (K) and protein (L) levels of SYT7 were analysed. Right, quantification of SYT7 level. (M, N) After cells were treated with or without 10 µM OGG1‐IN‐08 for 24 h, the mRNA (M) and protein (N) levels of SYT7 were analysed. Right, quantification of SYT7 level. All of the data are expressed as mean values ± SEM (*n* = 3); **p < 0.01, ***p < 0.001. (Student's *t*‐test).

It is well established that OGG1 functions as a transcriptional coactivator by binding to 8‐oxoG in the promoter region of target gene under oxidative stress (Pan et al., 2023, 2016; Wang et al., 2021). Defining the appropriate candidate target genes might explain the detailed mechanism by which OGG1 regulates EVs release. Our previous study has revealed that Ogg1 participated in insulin secretion through regulating Syt7 transcription in mouse models (Zhao et al., 2023). Considering that human SYT7 facilitates the exocytosis of dense nuclear vesicles during the fusion of endocrine cells and lysosomes (Liu et al., 2019), therefore, SYT7 might be the ideal candidate molecule mediating OGG1‐regulated EVs release. To test this hypothesis, we first confirmed this transcriptional regulation of SYT7 by OGG1 in human cells by OGG1 knockdown and knockout (Figures 3A–E and S3A). Given the necessity of 8‐oxoG in active gene promoter for OGG1's transcriptional regulatory function, we analysed the GC content of the 2 kb genomic sequence upstream of the SYT7 transcription start site (TSS) using the online CpG prediction tool (https://www.bioinformatics.nl/cgi‐bin/emboss/cpgplot). The analysis revealed a notably high GC content within the −844 to −55 nucleotide region (Figure 3F), consistent with the typical structural characteristics of genes regulated by OGG1. Next, we compared the mRNA and protein level of SYT7 before and after H_2_O_2_ treatment, and a significant upregulation was observed in the oxidative stress group (Figures 3G,H and S3B,C). Remarkably, this upregulation was inhibited by OGG1 knockdown or NAC exposure (Figure 3I,J). Likewise, the luciferase reporter gene assay confirmed that SYT7 expression was regulated by oxidation signal (Figure S3D,E). Further, to address the role of different enzymatic activities of OGG1 on SYT7 transcriptional regulation, the substrate binding activity inhibitor (Th5487) and enzyme cleavage activity inhibitor (OGG1‐IN‐08) of OGG1 were applied in the measurement. As expected, Th5487 pretreatment downregulated SYT7 expression (Figures 3K,L and S3F), whereas intriguingly, OGG1‐IN‐08 pretreatment did the opposite (Figure 3M,N). This suggested the importance of OGG1's DNA‐binding activity and the non‐necessity of enzymatic cleavage activity for its function in transcriptional regulation, which is consistent with the opinion that the binding of OGG1, bereft of its enzymatic cleavage activity, to DNA, thereby directs base excision repair towards gene transcription (Hao et al., 2020). Together, these data indicated that OGG1 relied on its substrate‐binding activity to regulate SYT7 transcription.

### 3.4 OGG1 promotes the binding of NF‐κB to SYT7 promoter region

**FIGURE 4:**
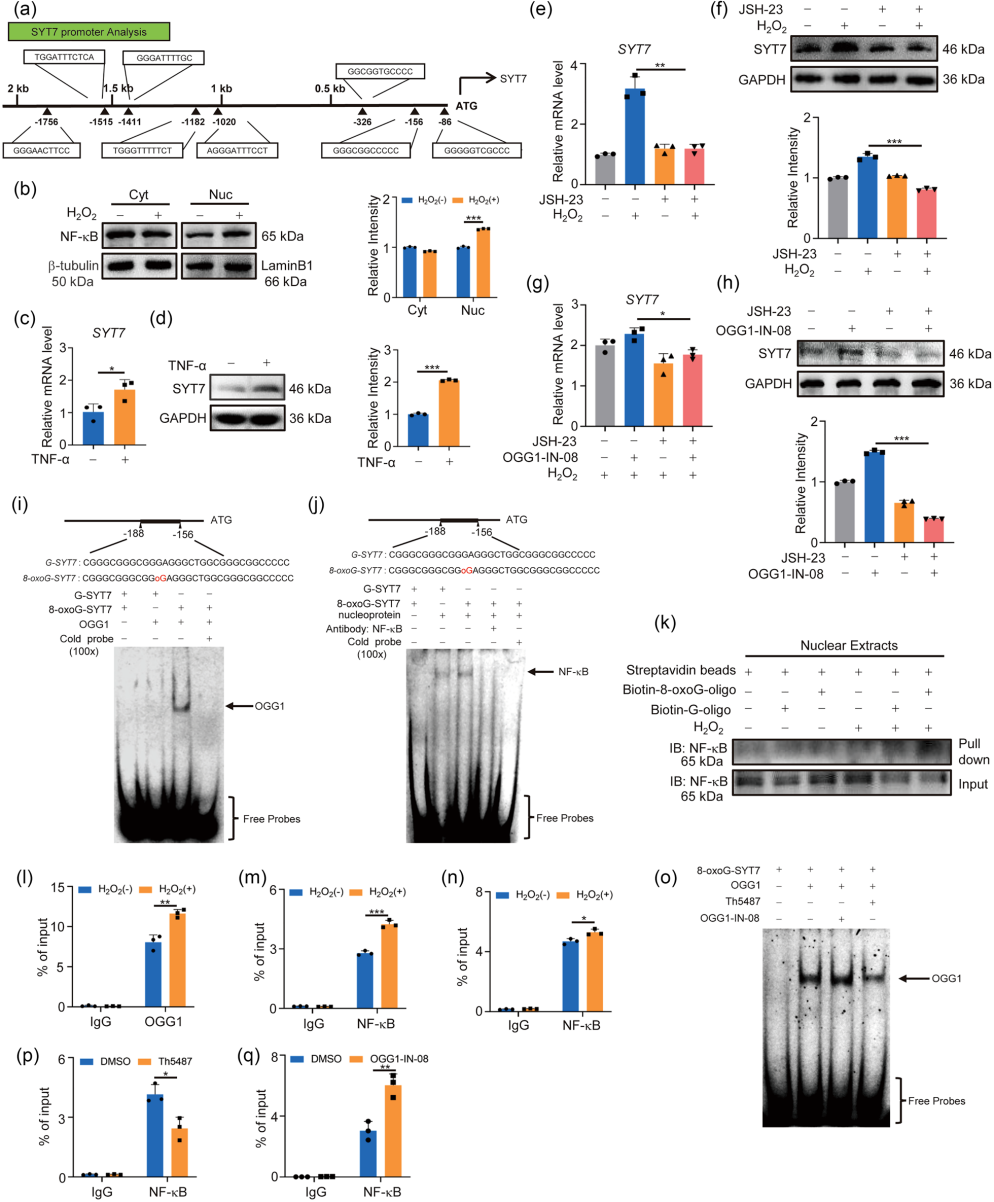
OGG1 promotes the binding of NF‐κB to target sites on the SYT7 promoter. (A) Analysis of the SYT7 promoter. NF‐κB binding sites in the SYT7 promoter region were predicted by JASPAR database. (B) The NF‐κB level in the cytoplasm and nucleus was analysed by Western blotting. Right, quantification of NF‐κB level. (C, D) A549 cells were exposed to 20 ng/mL TNF‐α for 4 h, and the mRNA (C) and protein (D) levels were analysed. Right, quantification of SYT7 level. (E, F) Cells were treated with or without 10 µM JSH‐23, and the mRNA (E) and protein (F) levels of SYT7 were analysed. Right, quantification of SYT7 level. (G, H) Cells were treated with or without 10 µM JSH‐23 or/and 10 µM OGG1‐IN‐08. The mRNA (G) and protein (H) levels of SYT7 were analysed. Right, quantification of SYT7 level. (I, J) 8‐oxoG increased the occupancy of OGG1 (I) or NF‐κB (J) on the SYT7 promoter. Purified OGG1 protein or nucleoprotein was subjected to EMSA with different FAM‐labelled probes. The arrow indicated the position of the NF‐κB or OGG1‐probe complex. (K) Occupancy of NF‐κB on 8‐oxoG‐containing DNA in nuclear extracts. Nuclear extracts (50 µg per sample) were incubated with G or 8‐oxoG probes for 12 h, and the protein‐DNA complexes were analysed by Western blotting. (L, M) ChIP‐qPCR assay determined the H_2_O_2_‐induced enrichment of OGG1 (L) or NF‐κB (M) on the SYT7 promoter. (N) ChIP‐qPCR detected the enrichment of NF‐κB at the SYT7 promoter in WT or OGG1‐KO cells under oxidative stress. (O) EMSA detected the effect of OGG1‐IN‐08 and Th5487 exposure on OGG1 binding to oxidised DNA strands. 10 µM OGG1‐IN‐08 or 10 µM Th5487 was incubated with 100 ng OGG1. (P) ChIP‐qPCR analysis of NF‐κB enrichment at the SYT7 promoter under oxidative stress with or without Th5487 treatment. (Q) ChIP‐qPCR analysis of NF‐κB enrichment at the SYT7 promoter under oxidative stress with or without OGG1‐IN‐08 treatment. All of the data are expressed as mean values ± SEM (*n* = 3); *p < 0.05, **p < 0.01, ***p < 0.001. (Student's *t*‐test).

As a transcriptional coactivator, OGG1's function depends on the recruitment of transcription factors. To investigate which transcription factors that synergise with OGG1 to regulate SYT7 expression, the SYT7 promoter was analysed using JASPAR (https://jaspar.genereg.net/), and multiple potential NF‐κB binding sites were predicted in SYT7 promoter (Figure 4A). Considering that NF‐κB functions in the nucleus, fluorescence localisation and nucleocytoplasmic separation analysis confirmed that oxidative stress exposure increased the distribution of NF‐κB and phospho‐NF‐κB in the nucleus, implying the activation of NF‐κB (Figures 4B and S4A). We also treated the cells with TNF‐α, the classic cytokine responsible for NF‐κB activation (Wamsley et al., 2015), to explore the potential involvement of NF‐κB in SYT7 expression. As anticipated, TNF‐α treatment increased the expression of SYT7 (Figure 4C,D). Conversely, restriction of NF‐κB subunit p65 entry into the nucleus by JSH‐23 resulted in a marked downregulation of SYT7 (Figures 4E,F and S4B). These results confirmed that SYT7 was a downstream gene of NF‐κB. Next, we assessed the importance of NF‐κB activation in the transcriptional regulation of SYT7 by OGG1. Since SYT7 expression has been shown to increase with OGG1‐IN‐08 treatment (Figure 3M,N), we examined whether such increase could be blocked after inhibition of NF‐κB nuclear import by JSH‐23. The reduced SYT7 expression with JSH‐23 exposure implied that the indispensability of NF‐κB for OGG1's involvement in SYT7 transcriptional regulation (Figures 4G,H and S4B). Then, to determine whether OGG1 and NF‐κB can bind the target sequence of SYT7, FAM‐labelled unoxidised (G‐SYT7) and oxidised probe (8‐oxoG‐SYT7) were synthesised for EMSA. The results revealed the specific binding of recombinant OGG1 protein to the oxidized probe (Figure 4I). Subsequently, the EMSA data with nuclear extracts showed that NF‐κB preferred binding to the oxidized probes, and the missing probe‐protein strip with anti‐NF‐κB antibody addition indicated the formation of a larger complex (Figure 4J). Likewise, more NF‐κB was pulled down by the biotin‐labelled oxidized oligos (Figure 4K). Furthermore, ChIP‐qPCR assay confirmed this binding in cells by showing the enhanced enrichment of both OGG1 and NF‐κB within the SYT7 promoter under oxidative stress (Figure 4L,M). Notably, this enrichment of NF‐κB was slightly increased (∼1.1 fold) in OGG1‐knockout cells (Figure 4N), which is less than that (∼1.6 fold) in WT cells, highlighting the crucial role of OGG1 in NF‐κB activation. Moreover, to explore the impact of OGG1's enzymatic activity on NF‐κB enrichment, cells were treated with two OGG1 inhibitors (OGG1‐IN‐08 and Th5487). With the different inhibitory effects, Th5487 and OGG1‐IN‐08 exhibited the inhibited and increased effects of OGG1 binding to oxidized DNA strands respectively (Figure 4O). The opposite effects on NF‐κB enrichment were also observed in H_2_O_2_‐treated A549 cells (Figure 4P,Q). These results strongly validated that OGG1, acting as an oxidized DNA sensor, mediated the binding of NF‐κB to the SYT7 promoter region. Collectively, these studies provided compelling evidence supporting the role of OGG1‐NF‐κB as a redox factor imperative for SYT7 expression.

### 3.5 SYT7 regulates EVs release

**FIGURE 5:**
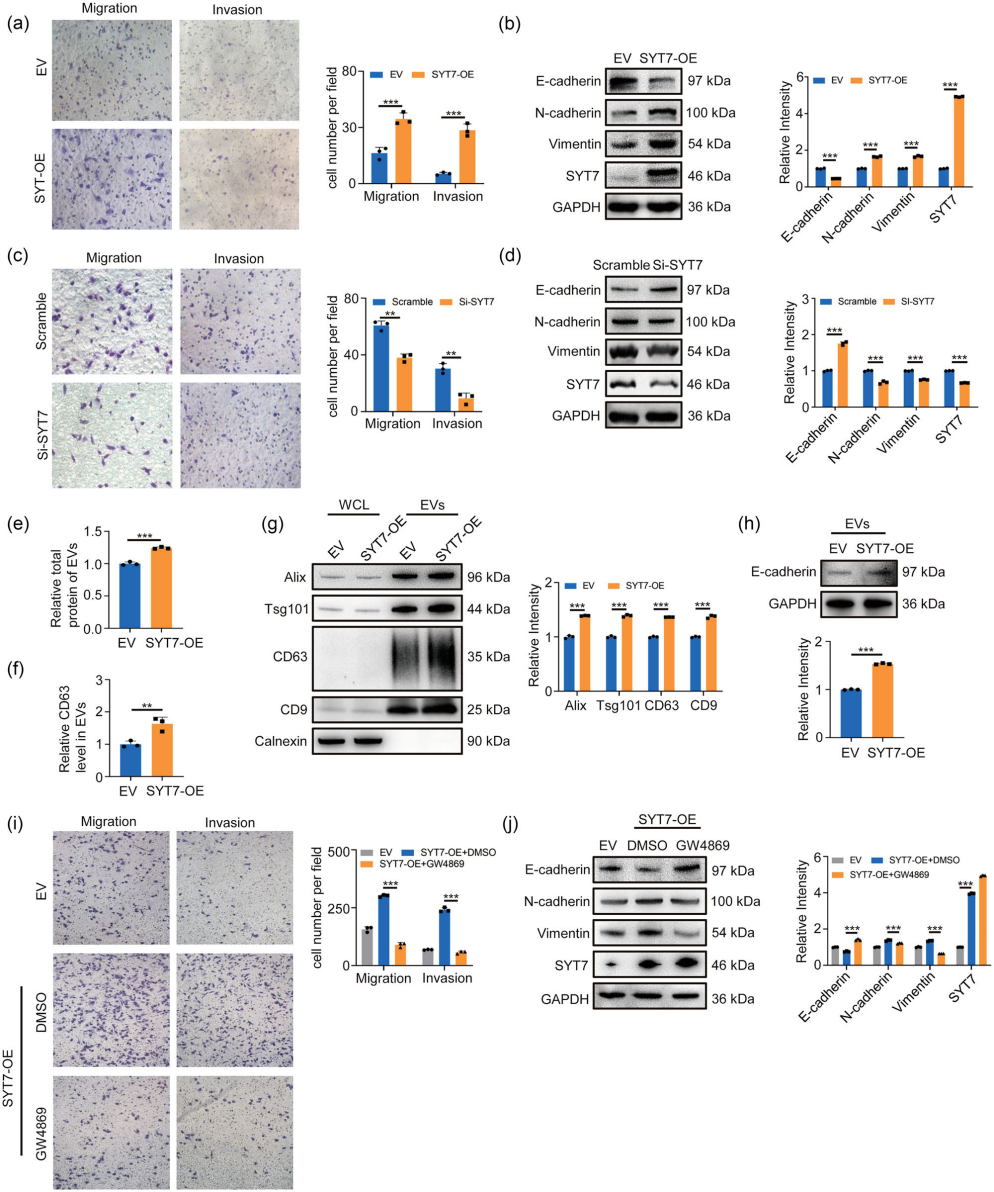
SYT7 reduces intracellular E‐cadherin by increasing EVs release. (A) Transwell assay was used to measure the migration of SYT7 overexpressed A549 cells. Right, quantification of migrated cells. (B) Western blotting analysis of EMT markers in SYT7 overexpressed A549 cells. Right, quantification of the marks. (C) SYT7 was knocked down by siRNA in A549 cells. After 24 h, the cells were digested and used in the transwell assay. Right, quantification of migrated cells. (D) Cells were treated as panel (C). Western blotting was used to measure EMT markers. Right, quantification of the marks. (E) The total protein concentration of isolated EVs was determined by BCA assay. (F) ELISA determined the content of CD63 in isolated EVs. (G) Western blotting analysed equal amounts of cell‐isolated EVs for markers content. Right, quantitative analysis of markers. (H) The content of E‐cadherin in equal amounts of EVs was quantified by Western blotting. (I) A549 cells were treated with or without 10 µM GW4869 for 24 h. Crystal violet staining for migrated cells. Right, quantification of migrated cells. (J) Cells were treated as panel (I). Western blot analysis was used to measure the level of EMT markers. Right, quantification of the marks. All of the data are expressed as mean values ± SEM (*n* = 3); **p < 0.01, ***p < 0.001. (Student's *t*‐test).

SYT7 is highly expressed in a variety of tumour tissues, contributing to a shortened disease‐free survival interval for patients (Figure S5A–E), implicating its involvement in tumour initiation and progression. Here, we found that SYT7 overexpression significantly enhanced cell migration, invasion and proliferation and altered EMT markers expression (Figures 5A,B and S5F,G), while SYT7 knockdown led to the opposite results (Figures 5C,D and S5H,I). It is well‐known that SYT7 is expressed on the lysosomal membrane and related to the lysosomal exocytosis (Martinez et al., 2000; Sung et al., 2015). The increased protein amount and marker protein levels of EVs by SYT7 overexpression confirmed that SYT7 regulated EVs release (Figures 5E–G and S5J,K). Notably, E‐cadherin level in EVs was accordingly increased (Figure 5H), providing a mechanistic explanation for SYT7 promoting tumour migration and invasion. To strengthen this result, the exosome release inhibitor GW4869 was used in SYT7‐overexpressed A549 cells, and the data showed that GW4869 diminished SYT7‐induced effects on cell migration, invasion and proliferation (Figures 5I,J and S5L). Altogether, these results suggested that SYT7 reduced intracellular E‐cadherin by increasing EVs secretion, thereby promoting cell migration and invasion.

### 3.6 SYT7 is involved in OGG1‐mediated tumour metastasis via EVs release

**FIGURE 6:**
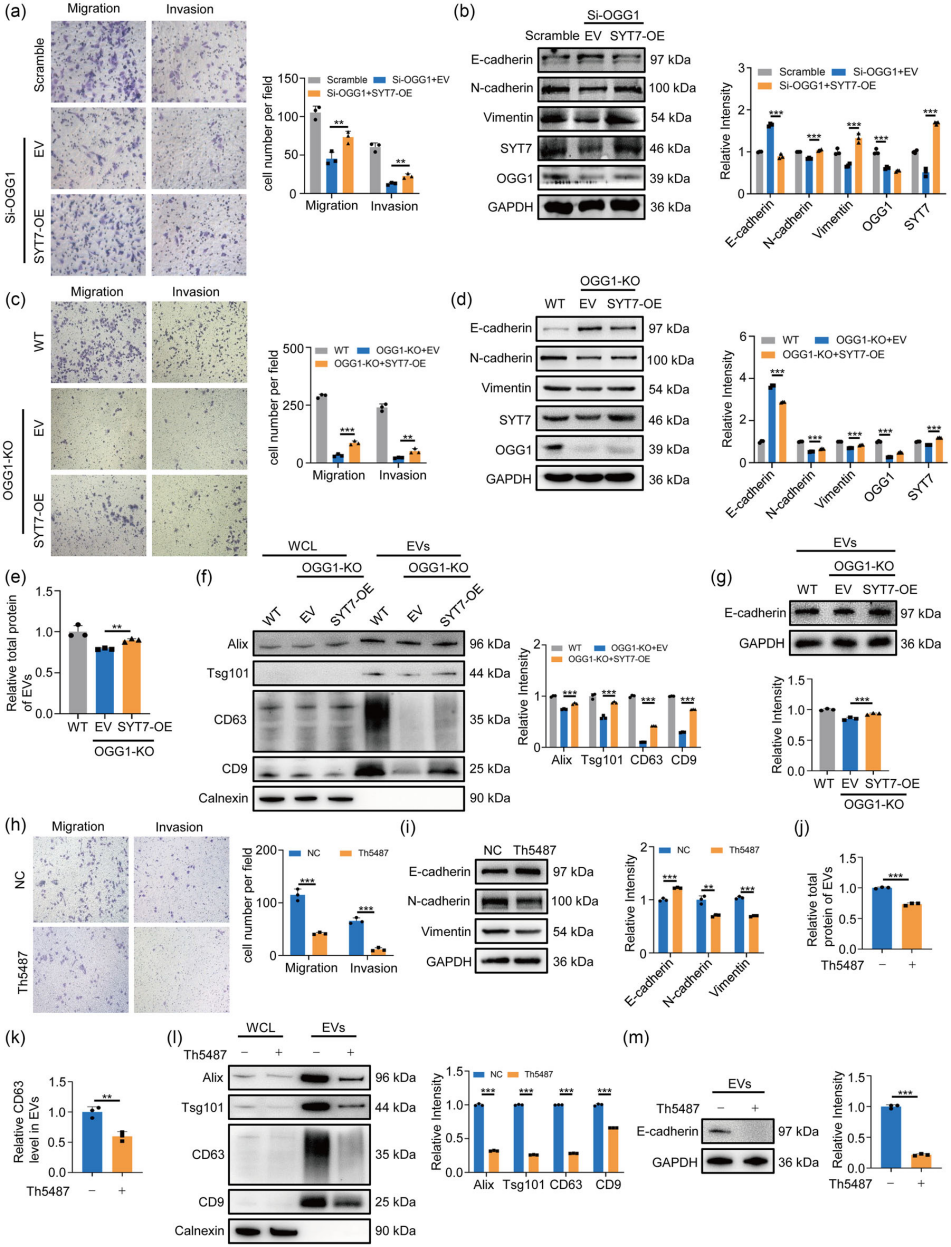
Overexpression of SYT7 partially restores the down‐regulation of migration caused by OGG1 deletion. (A) After overexpressing SYT7 in cells knocked down OGG1, transwell assay was used to detect migrated cells. Right, quantification of migrated cells. (B) Cells were treated as panel (A). Protein levels of EMT markers were verified by western blot assays. Right, quantitative analysis. (C) After overexpressing SYT7 in cells knocked out OGG1, transwell assay was used to detect migrated cells. Right, quantification of migrated cells. (D) Cells were treated as panel (C). Protein levels of EMT markers were verified by western blot assays. Right, quantitative analysis. (E) The total protein concentration of isolated EVs was determined by BCA assay. (F) Western blotting analysed equal amounts of cell‐isolated EVs for markers content. Right, quantitative analysis of markers. (G) The content of E‐cadherin in equal amounts of EVs was quantified by Western blotting. (H) Transwell assay detected the invasion and migration ability of A549 cells after Th5487 exposure. Right, quantification of migrated cells. (I) Cells were treated with Th5487 for 24 h. EMT‐related markers were detected by Western blotting. Right, quantification of the marks. (J) After cells were treated with Th5487 for 24 h, EVs from the culture supernatant were isolated by ultra‐fractionation. The total protein concentration of isolated EVs was determined by BCA assay. (K) ELISA determined the content of CD63 in isolated EVs. (L) Western blotting analysed equal amounts of cell‐isolated EVs for markers content. Right, quantitative analysis of markers. (M) The content of E‐cadherin in equal amounts of EVs was quantified by Western blotting. All of the data are expressed as mean values ± SEM (*n* = 3); **p < 0.01, ***p < 0.001. (Student's *t*‐test).

Previous results have verified the transcriptional regulation of SYT7 by OGG1. Thus, to validate the function of OGG1/SYT7 axis in tumour metastasis, we first expressed SYT7 in OGG1‐knockdown or OGG1‐knockout cells. The data showed that OGG1‐deficiency‐suppressed cancer cell migration, invasion and proliferation were rescued by SYT7 overexpression (Figures 6A–D and S6A). Furthermore, the overexpressed SYT7 also partially restored EVs secretion and E‐cadherin level in EVs (Figure 6E–G). Taken together, these results confirmed that OGG1/SYT7 axis was involved in cell migration and invasion by regulation of EVs release. Next, we used OGG1 substrate binding inhibitor Th5487 to investigate the role of OGG1 blocking on cell metastasis. We found that Th5487 treatment remarkably reduced cell migration, invasion, proliferation and EMT (Figures 6H,I and S6B). Furthermore, Th5487 treatment also decreased EVs release (Figures 6J–L and S6C,D) and E‐cadherin levels in EVs (Figure 6M). These results revealed that OGG1 inhibitor mitigated the loss of intracellular E‐cadherin by suppressing the release of EVs and reducing cell metastasis.

### 3.7 Reduction of OGG1 binding to DNA represses lung cancer metastasis

**FIGURE 7:**
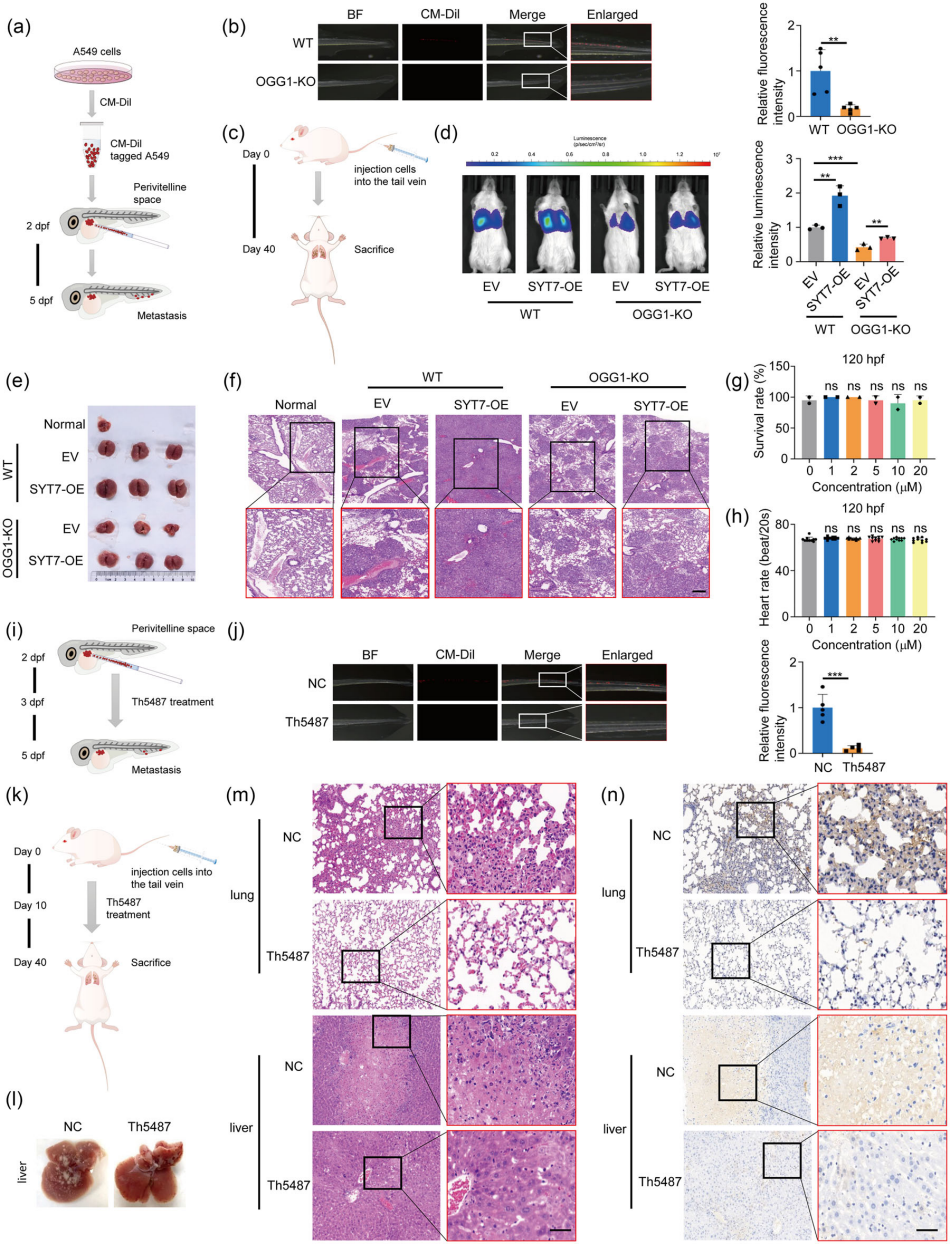
OGG1 deletion reduces lung cancer metastasis in vivo. (A) Schematic diagram of the experimental process of zebrafish xenograft. CD‐Dil‐labelled A549 cells (red) were injected into the perivitelline space of each zebrafish. (B) A zebrafish xenograft model was used to evaluate the metastatic ability of A549 cells after OGG1 knockout. Representative images of zebrafish were shown. (C) Schematic diagram of establishing a lung cancer metastasis model by injecting A549 cells into the tail vein. (D) In vivo bioluminescence imaging of mice 40 days after cell injection. (E) Gross anatomy of mouse lungs. (F) Representative HE staining images of lungs from each group. Scale bar: 200 µm. (G, H) Zebrafish juvenile survival rate (G) and heart rate (H) at 120 hpf. Conventional one‐way *ANOVA* test was performed considering the experimental group as the independent variable. ns stands for no significance. (I) Schematic diagram of an experimental zebrafish xenograft experiment using A549 cells to test the anti‐tumour effect of Th5487. (J) Zebrafish xenograft model was used to evaluate the effect of Th5487 on A549 cell metastasis. Representative images of zebrafish were shown. (K) A mouse lung metastasis model was established by injecting A549 cells into the tail vein. Ten days after cell injection, mice were intraperitoneally injected with 30 mg/kg Th5487 or a corresponding volume of PBS every 2 days. (L) Gross anatomy of mouse liver. (M) Representative HE staining images of lungs and liver in each group. Scale bar: 50 µm. (N) Representative images from IHC staining for SYT7. Scale bar: 50 µm. All of the data except zebrafish juvenile survival rate and heart rate are expressed as mean values ± SEM (*n* = 3); **p < 0.01, ***p < 0.001. (Student's *t*‐test).

To delve deeper into the impact of OGG1/SYT7 axis on cancer metastasis in vivo, we first employed a zebrafish xenograft model, which allows quick analyses of cancer progression (Figure 7A). CM‐Dil‐tagged WT or OGG1‐KO cells were microinjected into the middle of the zebrafish yolk sac near the dorsal side and after 3 days, five zebrafish were randomly selected for analysis. It showed that OGG1 knockout significantly reduced tumour metastasis after 5 days postfertilisation (dpf) (Figure 7B). Next, we established xenograft murine model of lung cancer by injecting A549 cells stably expressing luciferase (A549‐luc) into the tail vein (Figure 7C). In vivo fluorescence imaging was performed 40 days after injection to observe tumour metastasis. Biofluorescence imaging (BLI) quantification showed that SYT7 overexpression significantly increased lung tumour formation, whereas OGG1 knockout exerted the opposite effect (Figure 7D). Notably, the effect of OGG1 knockout on tumour metastasis can be rescued by SYT7 overexpression. The burden of mouse lung tumour was also depicted in Figure 7E. Furthermore, the results of haematoxylin‐eosin (HE) staining further indicated that overexpression of SYT7 resulted in more metastatic nodules (Figures 7F and S7A). Since Figure 6H demonstrated the significant inhibition of cell migration ability by Th5487, we subsequently explored whether Th5487 could be applied to inhibit tumour metastasis in vivo. After testing the maximum tolerated concentration (MTC) of Th5487 in the zebrafish model (Figures 7G,H and S7B–E), 20 µM Th5487 was selected for zebrafish xenograft efficacy experiments (Figure 7I), and the data demonstrated that Th5487 treatment exhibited a significant inhibitory effect on tumour metastasis (Figure 7J). Similarly, we evaluated the effect of Th5487 on tumour metastasis in mouse models (Figure 7K). As expected, Th5487 treatment significantly alleviated the tumour burden (Figure 7L) and reduced tumour metastasis (Figure 7M). The results of immunohistochemistry (IHC) staining further confirmed the elevated expression of SYT7 in tumour tissues (Figure 7N). Collectively, these data suggested that OGG1/SYT7 axis played a crucial role in tumour metastasis, which could be effectively suppressed by OGG1 inhibitor.

## 4 DISCUSSION

**FIGURE 8:**
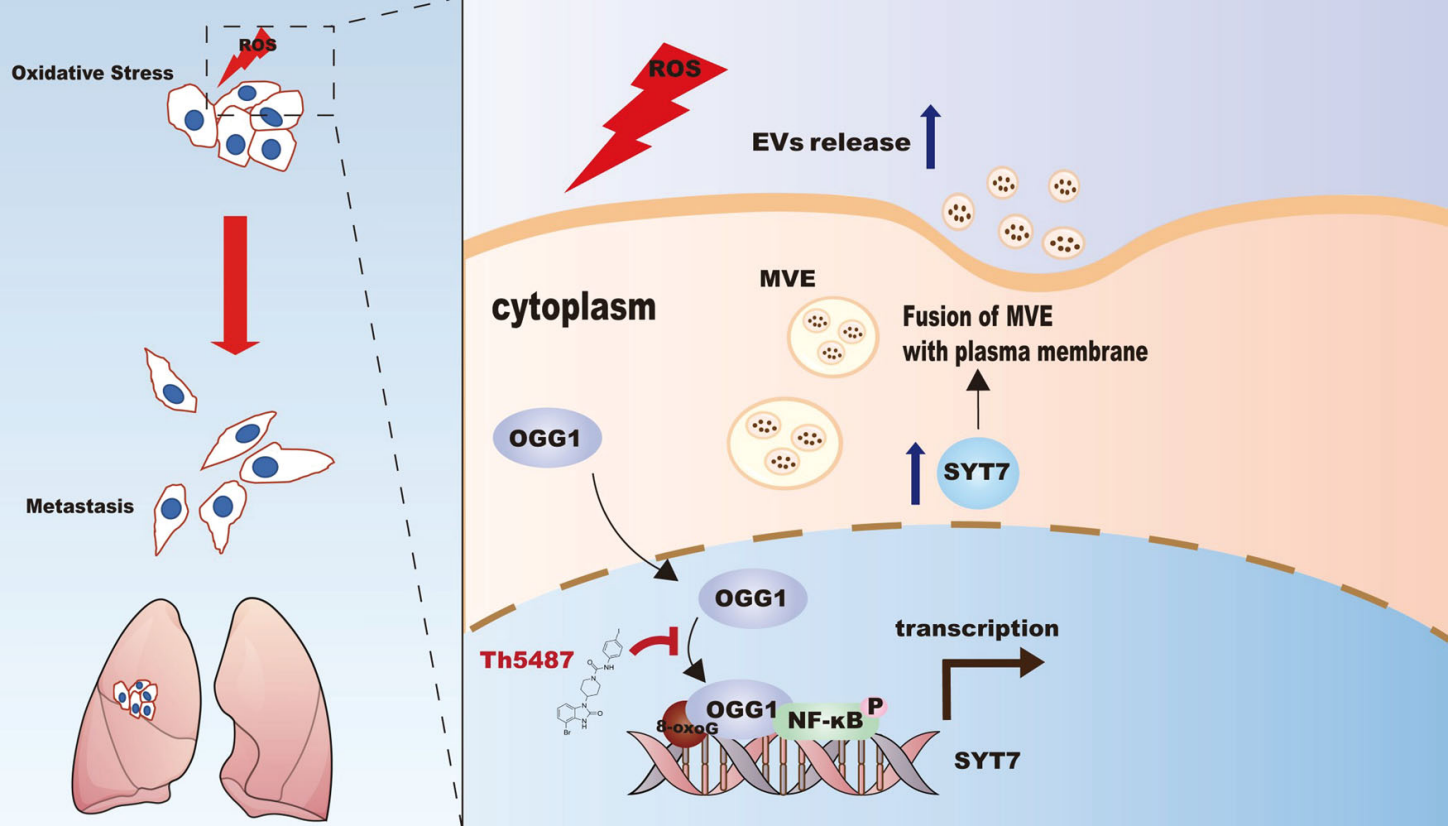
Schematic diagram of OGG1 affecting SYT7 expression to mediate EVs release. This model shows that under oxidative stress, OGG1 increases the accessibility of NF‐κB in the SYT7 promoter region, upregulates its expression, further promotes the secretion of EVs and participates in the EMT process.

Efficient DNA oxidative repair is indispensable for maintaining homeostasis, which is a process that tightly links DNA oxidative damage signalling and tumour development in vivo. In this process, 8‐oxoG acts as an intracellular signal to increase tumour migration due to the imbalance of redox homeostasis in cancer cells. Our biochemical and molecular studies have established a model in which base‐excision repair (BER) proteins initiate transcription and participate in EVs secretion, further promoting tumour metastasis. Specifically, OGG1 binds to 8‐oxoG and synergises with the transcription factor NF‐κB to activate SYT7 transcription. Subsequently, upregulated SYT7 increased the release of EVs loaded with more E‐cadherin. The cumulative effect of these interactions ultimately determined the response of cancer cells to oxidative stress. These results, together with the mechanism in Figure 8, provide novel insights into 8‐oxoG/OGG1‐initiated EVs release under oxidative stress, highlighting its role as a catalyst for tumour migration.

EVs have become a key player in intercellular communication and are closely related to various stages of tumourigenesis and development (Kalluri & McAndrews, 2023). Consistent with these conclusions, our results showed that inhibiting exosome release with GW4869 effectively reduced oxidative stress‐induced tumour cell metastasis. The release of EVs is regulated by various factors, such as hypoxia, lactic acid, phosphatidylinositol switch, extracellular matrix (Liu et al., 2023; Wu et al., 2023; Xi et al., 2021; Yang et al., 2022). In this study, we observed that the oxidised DNA signal also played a key role in EVs release. Once the oxidative stress signal was blocked by knocking down/knocking out of OGG1, the sensor for oxidative DNA, or by inhibiting OGG1‐DNA binding, EVs release was significantly inhibited. Based on these findings, we propose a novel pathway by which OGG1 reads oxidative DNA signals and promotes EVs secretion and subsequent tumour metastasis.

Tumour metastasis is the result of multiple factors. Among them, transcription factor family, such as SNAIL and TWIST, are well known to be up‐regulated by multiple EMT‐inducers, inhibit E‐cadherin expression and subsequently initiate tumour metastasis (Fan et al., 2022). The increased expression of SNAIL1 and TWIST1 under oxidative stress was detected by our and previous studies (Jung et al., 2016; Liu et al., 2018), confirming their function in oxidative stress‐induced metastasis. Notably, we found that knocking down or knocking out of OGG1 significantly inhibited the protein levels of SNAIL1 and TWIST1, suggesting that these transcription factors may be regulated by OGG1. This result was supported by OGG1 overexpression‐increased SNAIL1 protein level in the study of lung fibrosis (Song et al., 2024). As the sensor for oxidative DNA, OGG1 was involved in cellular response upon oxidative stress through its transcriptional regulation of downstream genes (Geng et al., 2024; Pan et al., 2023; Song et al., 2017, 2024). It is possible that 8‐oxoG/OGG1 could drive tumour metastasis through induction of not only SYT7, but also SNAIL1 and TWIST1. Interestingly, it has been reported that TWIST1 is the transcriptional factor of SYT7 (Liu et al., 2019). Therefore, the possibility may also exist that OGG1 recruits TWIST1 to regulate SYT7 transcription and SYT7‐mediated EVs release, which needs further investigation in future work. Taken together, our results addressed the initial signal function of 8‐oxoG/OGG1 in upstream regulation of metastasis.

SYT7, a member of the synaptotagmin family, is expressed on the lysosomal membrane of a variety of cells, and induces Ca^2+^‐triggered lysosomal exocytosis and plasma membrane repair through the C2 domain (Martinez et al., 2000). SYT7 also interacts with SNARE complex components Syntaxin‐1A (STX1A) and Syntaxin‐3 (STX3) through the C2 domain in a Ca^2+^‐dependent manner to promote the exocytosis of insulin by human pancreatic β cells (Dolai et al., 2016). Surprisingly, recent studies have reported that SYT7 is highly expressed in cancers such as gastric cancer and osteosarcoma, and promotes cell proliferation and migration (Kanda et al., 2018; Liu et al., 2019; Wu et al., 2020). Consistent with these previous studies, SYT7 was highly expressed in lung cancer in the TCGA database. We observed that cell migration was significantly increased or decreased by SYT7 overexpression or knockdown, respectively, suggesting that SYT7 functions in cancer progression and could be a potential target for cancer therapy. As a membrane‐associated protein, SYT7 has been reported to involve in the secretion of EVs (Sung et al., 2015). Consistent with their results, we observed not only an upregulation of EVs release by SYT7, but also an increased loading of E‐cadherin in EVs. In a variety of tumour cells, a critical indicator of EMT is the downregulation of E‐cadherin expression. Abnormal expression of E‐cadherin destabilises the extracellular skeleton and basement membrane, weakens cell‐cell adhesion, enhances cell activity and metastasis, and makes tumours prone to metastasis. Up to date, several explanations have been proposed for the reduction of intracellular E‐cadherin (Li et al., 2017; Xue et al., 2019; Zhang et al., 2019). Here, we found that the reduced intracellular E‐cadherin levels were partially due to the loss of E‐cadherin that accompanies EVs release, as more E‐cadherin was detected in EVs. This mechanical explanation could be further confirmed by OGG1 knockdown and Th5487 exposure, both of which down‐regulated SYT7 expression, reduced EVs secretion and E‐cadherin load in released EVs. Moreover, Th5487 treatment repressed lung tumour development in mice, providing a plausible basis for clinical treatment to specifically weaken cancer metastasis.

In conclusion, we explored the mechanism of oxidative stress‐induced cancer cell metastasis and revealed that ROS‐mediated DNA oxidative damage is the key messenger initiating OGG1 and transcription factors to regulate gene expression, EVs release and tumour metastasis, which has important implications for understanding the response of cancer cells to the tumour microenvironment. These results strongly suggest that oxidative DNA damage and the repair protein are critical for tumour development, which could be the potential therapeutic targets for cancer treatment.

## AUTHOR CONTRIBUTIONS

Ying Ma: Conceptualization (equal); formal analysis (equal); funding acquisition (equal); investigation (equal); methodology (equal); supervision (equal); writing—original draft (equal); writing—review and editing (equal). Jiarong Guo: Writing—review and editing (supporting). Haipeng Rao: Formal analysis (supporting). Jingyu Xin: Investigation (supporting). Xinyi Song: Investigation (supporting); Rui Liu: Investigation (supporting), Shan Shao: Formal analysis (supporting). Jiajia Hou: Formal analysis (supporting). Liyu Kong: Formal analysis (supporting). Zhigang Hu: Formal analysis (supporting). Lingfeng He: Formal analysis (supporting). Feiyan Pan: Conceptualization (equal); funding acquisition (equal); methodology (equal); project administration (equal); supervision (equal); writing—original draft (equal); writing—review and editing (equal). Zhigang Guo: Conceptualization (equal); funding acquisition (lead); project administration (equal).

## CONFLICT OF INTEREST STATEMENT

The authors declare no conflict of interest.

## Supporting information

Supporting information

## Data Availability

The data that support the findings of this study are available from the corresponding author upon reasonable request.
