# Supplementary material for: The 8‐oxoguanine DNA glycosylase‐synaptotagmin 7 pathway increases extracellular vesicle release and promotes tumour metastasis during oxidative stress
Source: J Extracell Vesicles. 2024 Sep 5;13(9):e12505. doi: 10.1002/jev2.12505 (PMC11375530; doi:10.1002/jev2.12505)
Supplement: Supplementary file 1 — Supporting information [file JEV2-13-e12505-s001.docx]

**The 8-oxoguanine DNA glycosylase-synaptotagmin 7 pathway increases extracellular vesicle release and promotes tumor metastasis during oxidative stress**

Ying Ma^1^, Jiarong Guo^1^, Haipeng Rao^1^, Jingyu Xin^1^, Xinyi Song^1^, Rui Liu^1^, Shan Shao^1^, Jiajia Hou^1^, Liyu Kong^1^, Zhigang Hu^1^, Lingfeng He^1^, Feiyan Pan^1^* and Zhigang Guo^1^*

^1^ Jiangsu Key Laboratory for Molecular and Medical Biotechnology, College of Life Sciences, Nanjing Normal University, 1 Wen Yuan Road, Nanjing, 210023, China

* To whom correspondence should be addressed:

panfeiyan@njnu.edu.cn or guo@njnu.edu.cn

**Supporting Information**

The following file contains supplementary material for the paper “The 8-oxoguanine DNA glycosylase-synaptotagmin 7 pathway increases extracellular vesicle release and promotes tumor metastasis during oxidative stress”.

This file is composed of:

- Supplementary figures (7 figures)
- Supplementary table (1 table)

**
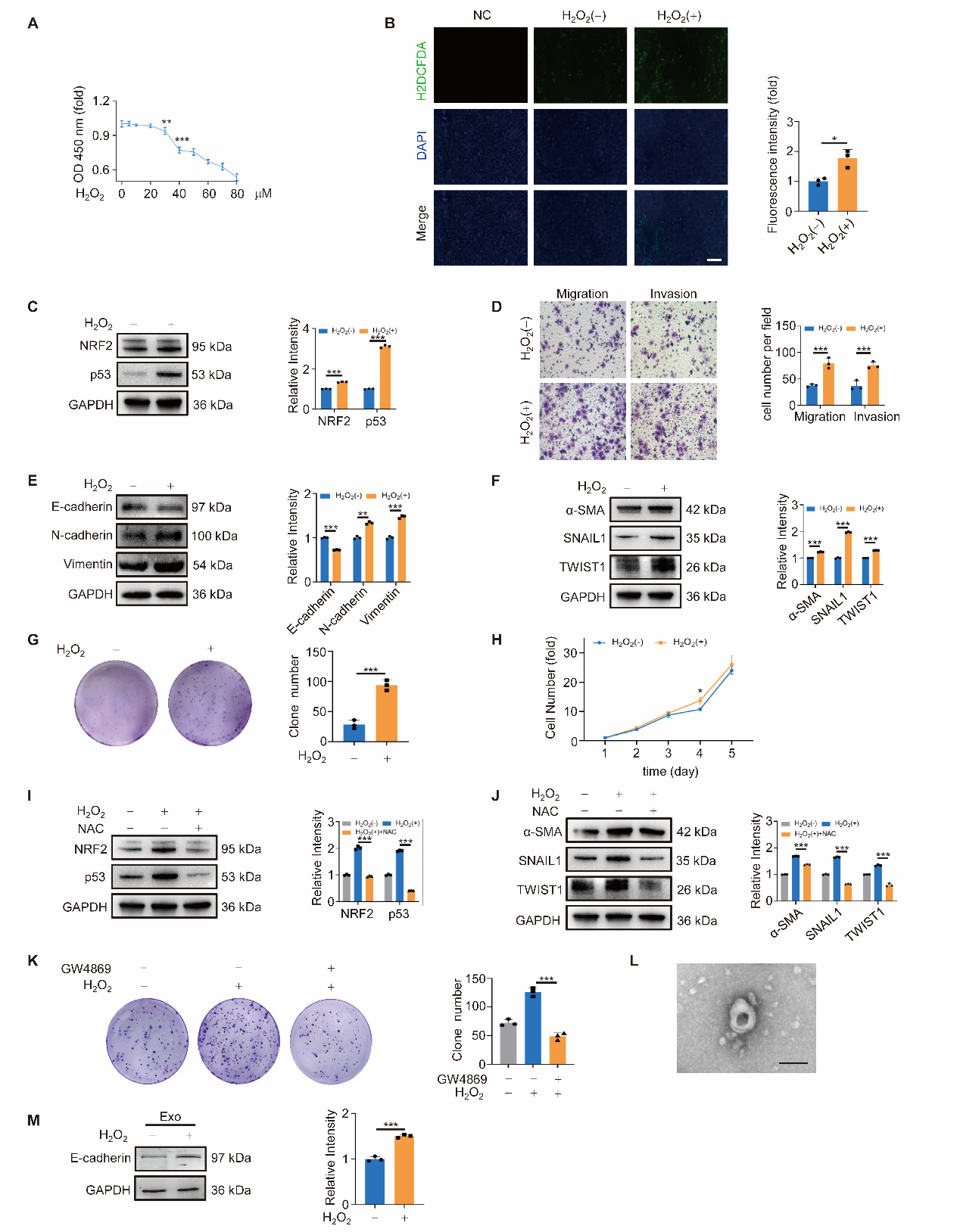
Figure S1 Oxidative stress increases migration and proliferation.** (**A**) The CCK8 assay was used to detect the effect of H_2_O_2_ concentration gradient for 48 hours on the viability of A549 cells. (**B**) Detection of intracellular ROS generation in A549 cells exposed to 20 μM H_2_O_2_ for 48 h. Scale bar: 100 μm. Right, quantification of corresponding fluorescence intensity. (**C**) A549 cells were treated with or without 20 μM H_2_O_2_ for 24 h. The protein levels of NRF2 and p53 were measured by Western blotting. Right, quantification of NRF2 and p53. (**D**) H460 cells were treated with or without 20 μM H_2_O_2_ for 24 h. Crystal violet staining for migrated cells. Right, quantification of migrated cells. (**E**) Western blotting analysis of EMT markers levels after 20 μM H_2_O_2_ exposure. Right, quantification of marks. (**F**) Cells were treated with or without 20 μM H_2_O_2_ for 24 h and analyzed by Western blotting. Right, quantification of markers. (**G**) The clonogenic ability of A549 cells after 20 μM H_2_O_2_ exposure was examined by colony formation assay. Right, quantification of colony numbers. (**H**) The CCK8 method was used to detect the growth of A549 cells after 20 μM H_2_O_2_ exposure. (**I**) A549 cells were treated with 20 μM H_2_O_2_ and 10 mM NAC for 24 h and analyzed by Western blotting. Right, quantification of NRF2 and p53. (**J**) Cells were treated as panel (**I**). Protein levels of α-SMA, SNAILl and TWIST1 were analyzed by Western blotting. Right, quantification of markers. (**K**) The clonogenic ability of A549 cells treated with or without GW4869 after 20 μM H_2_O_2_ exposure was examined by colony formation assay. Right, quantification of colony numbers. (**L**) EVs were photographed by transmission electron microscopy. Scale bar: 100 nm. (**M**) EVs were collected after treatment with 20 μM H_2_O_2_ for 24 h. Western blotting analysis of E-cadherin in equal amounts of EVs. GAPDH was used as a loading control. Right, quantification of E-cadherin. All of the data are expressed as mean values ± SEM (n = 3); ***P*<0.01, ****P*<0.001. (Student's *t*-test).


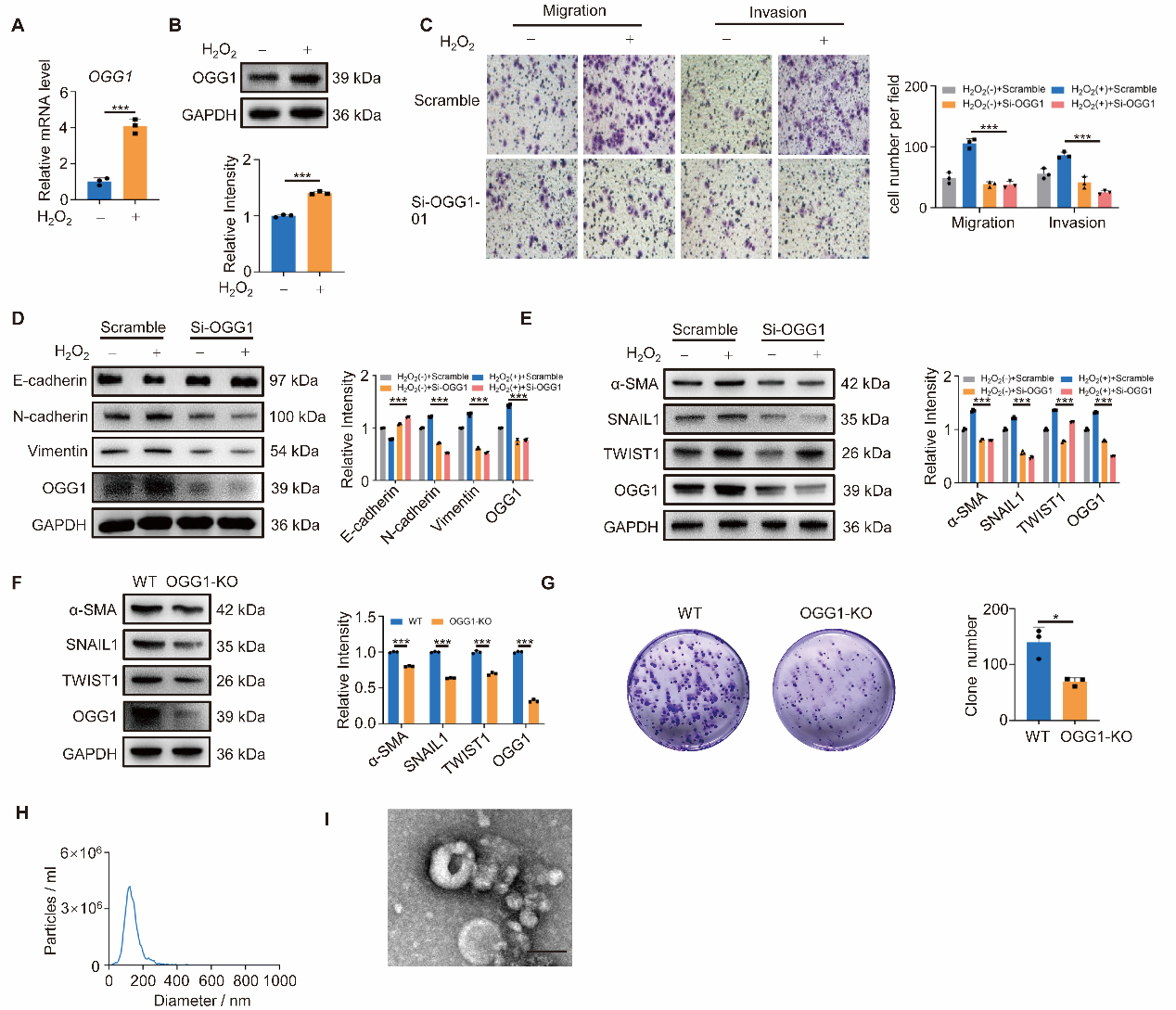
**Figure S2 OGG1 deletion reduces migration.** (**A, B**) H460 cells were treated with or without 20 μM H_2_O_2_ for 24 h. The mRNA (**A**) and protein (**B**) level of OGG1 were measured by RT-qPCR and Western blotting. Right, the quantization of OGG1. (**C**) OGG1 was knocked down by siRNA in H460 cells. After 24 h, the cells were digested and used in the transwell assay. Right, quantification of migrated cells. (**D**) Western blotting was used to measure EMT markers. Right, the quantification of markers. (**E**) Western blotting was used to measure the protein levels of α-SMA, SNAILl and TWIST1. Right, quantification of markers. (**F**) The protein levels of α-SMA, SNAILl and TWIST1 in WT and OGG1-KO cells was detected by western blotting. Right, quantification of markers. (**G**) The clonogenic ability of WT and OGG1-KO cells was examined by colony formation assay. Right, quantification of colony numbers. (**H**) The concentration and diameter of isolated EVs were detected by NTA after knocking out OGG1. (**I**) EVs were photographed by transmission electron microscopy after knocking out OGG1. Scale bar: 100 nm. All of the data are expressed as mean values ± SEM (n = 3); **P*<0.05, ****P*<0.001. (Student's *t*-test).

**
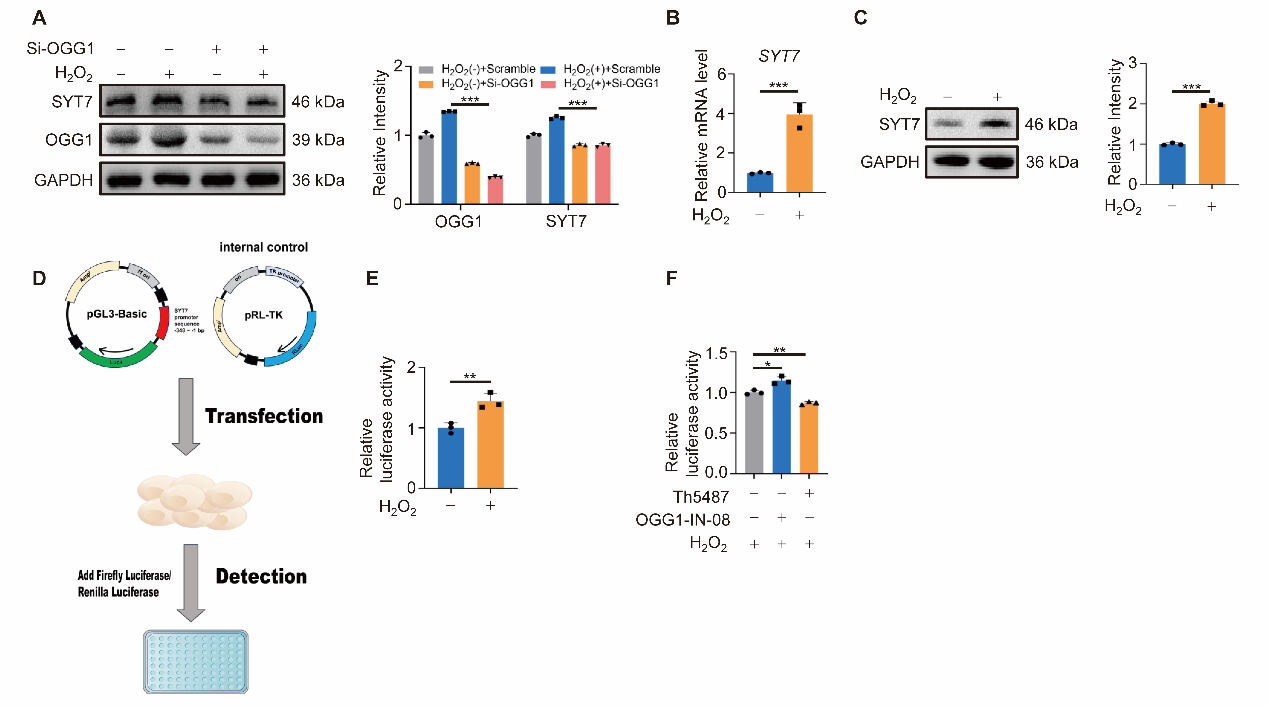
Figure S3 OGG1 deletion inhibits oxidative stress-induced upregulation of SYT7.** (**A**) Western blotting was used to determine the level of SYT7 in the H460 cells transfected with scrambled siRNA or OGG1 siRNA. Right, quantification of SYT7. (**B,** **C**) H460 cells were treated with or without 20 μM H_2_O_2_ for 24 h. The mRNA (**B**) and protein (**C**) level of SYT7 were measured by RT-qPCR and Western blotting. Right, the quantization of SYT7. (**D**) Schematic diagram of dual-luciferase reporter gene assay. Luciferase assay was performed using A549 cells co-transfected with a luciferase reporter plasmid (pGL3-basic) containing SYT7 promoter region -348 ~ -1 bp and a plasmid encoding renilla luciferase (pRL-TK). (**E**) After cells were treated with 20 μM H_2_O_2_, luciferase was detected. (**F**) Luciferase detection after cells were treated with or without Th5487 or OGG1-IN-08. All of the data are expressed as mean values ± SEM (n = 3); **P*<0.05, ***P*<0.01, ****P*<0.001. (Student's *t*-test).


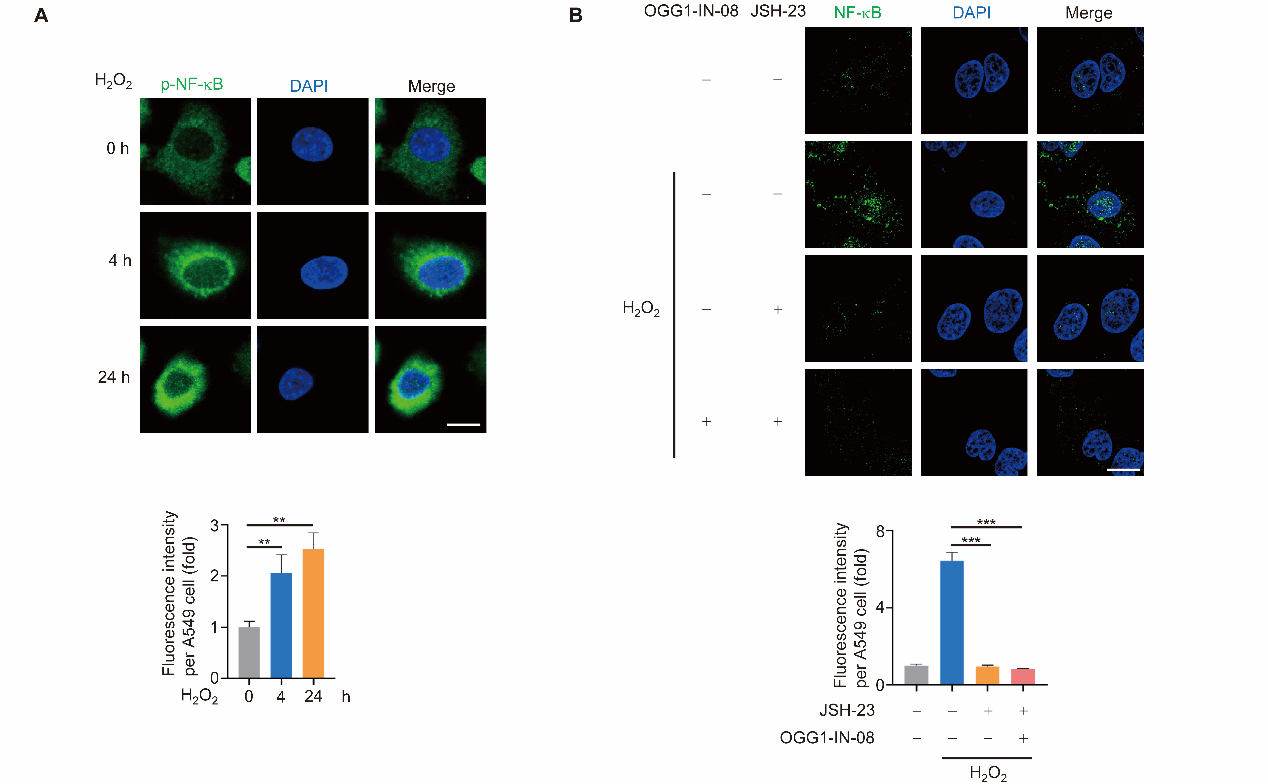


**Figure S4 Oxidative stress increases nuclear translocation of p-NF-κB.** (**A**) A549 cells were treated with 20 μM H_2_O_2_ and then subjected to IF for p-NF-κB (green) using confocal microscopy. DAPI (blue) was used to stain cell nuclei and display merged images. Scale bar: 10 µm. Below, the quantification of immunostaining for p-NF-κB. (**B**) Cells were treated with or without 10 μM JSH-23. The distribution of NF-κB (green) detected by IF. Scale bar: 10 μm. Below, the fluorescence intensity per A549 cell (fold) for NF-κB. All of the data are expressed as mean values ± SEM (n = 3); ***P*<0.01. (Student's *t*-test).


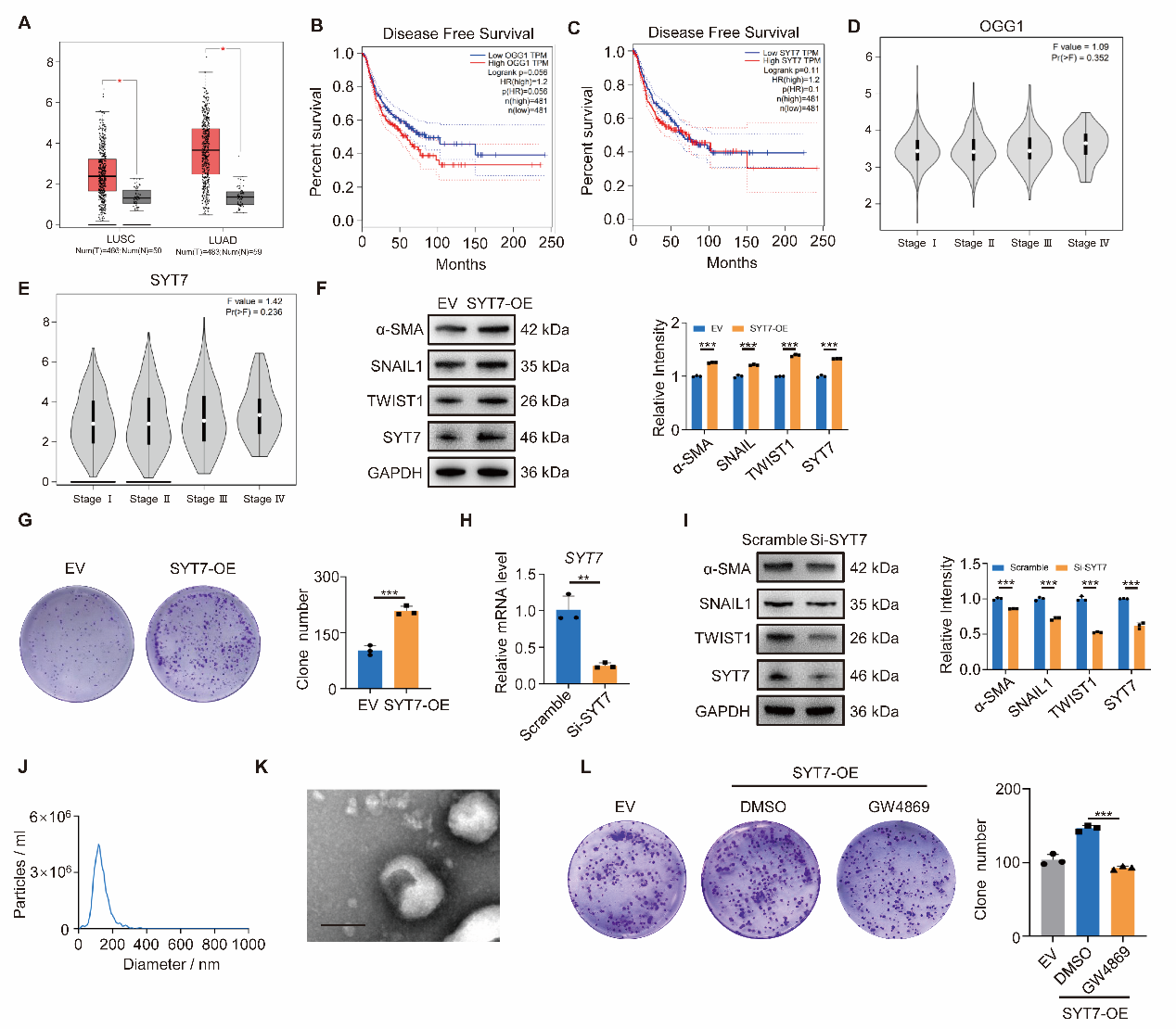
**Figure S5 SYT7 is highly expressed in lung cancer.** (**A**) Analysis of SYT7 expression in lung tumors in the GEPIA online database. (**B**) The disease-free survival cure of OGG1 in patients with LUSC and LUAD (GEPIA). (**C**) The disease-free survival cure of SYT7 in patients with LUSC and LUAD (GEPIA). (**D**) Stage diagram of OGG1 expression levels and pathological stages of LUSC and LUAD in the GEPIA database. (**E**) Stage diagram of SYT7 expression levels and pathological stages of LUSC and LUAD in the GEPIA database. (**F**) Western blotting analysis of α-SMA, SNAIL1 and TWIST1 in SYT7 overexpressed A549 cells. Right, quantification of the marks. (**G**) The clonogenic ability of SYT7 overexpressing cells was examined by colony formation assay. Right, quantification of colony numbers. (**H**) A549 cells transfected with scrambled siRNA or SYT7 siRNA. The mRNA level of SYT7 were measured by RT-qPCR. (**I**) Western blot analysis of α-SMA, SNAILl, and TWIST1 in SYT7 knockdown A549 cells. Right, quantification of the marks. (**J**) The concentration and diameter of isolated EVs were detected by NTA after overexpression of SYT7. (**K**) EVs were photographed by transmission electron microscopy after overexpression of SYT7. Scale bar: 100 nm. (**L**) The clonogenic ability of SYT7 overexpressing cells treated with or without GW4869 was examined by colony formation assay. Right, quantification of colony number. All of the data are expressed as mean values ± SEM (n = 3); ***P*<0.01, ****P*<0.001. (Student's *t*-test).


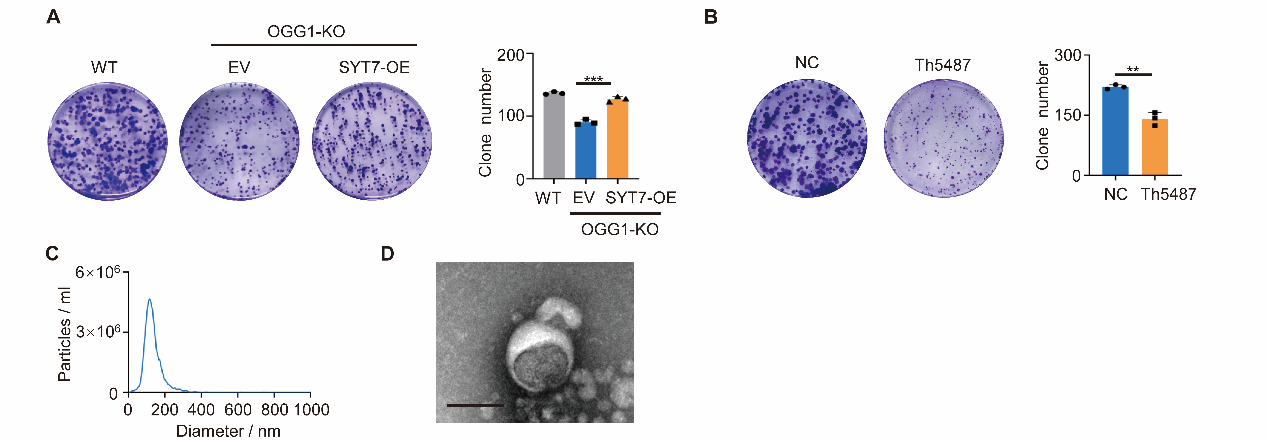


**Figure S6 Overexpression of SYT7 partially compensates for the down-regulation of proliferation caused by OGG1 loss.** (**A**) Representative colony formation images of A549 cells. (**B**) Representative colony formation images of A549 cells treated with or without Th5487. (**C**) The concentration and diameter of isolated EVs were detected by NTA after Th5487 treatment. (**D**) EVs were photographed by transmission electron microscopy after Th5487 treatment. Scale bar: 100 nm. All of the data are expressed as mean values ± SEM (n = 3); ***P*<0.01, ****P*<0.001. (Student's *t*-test).


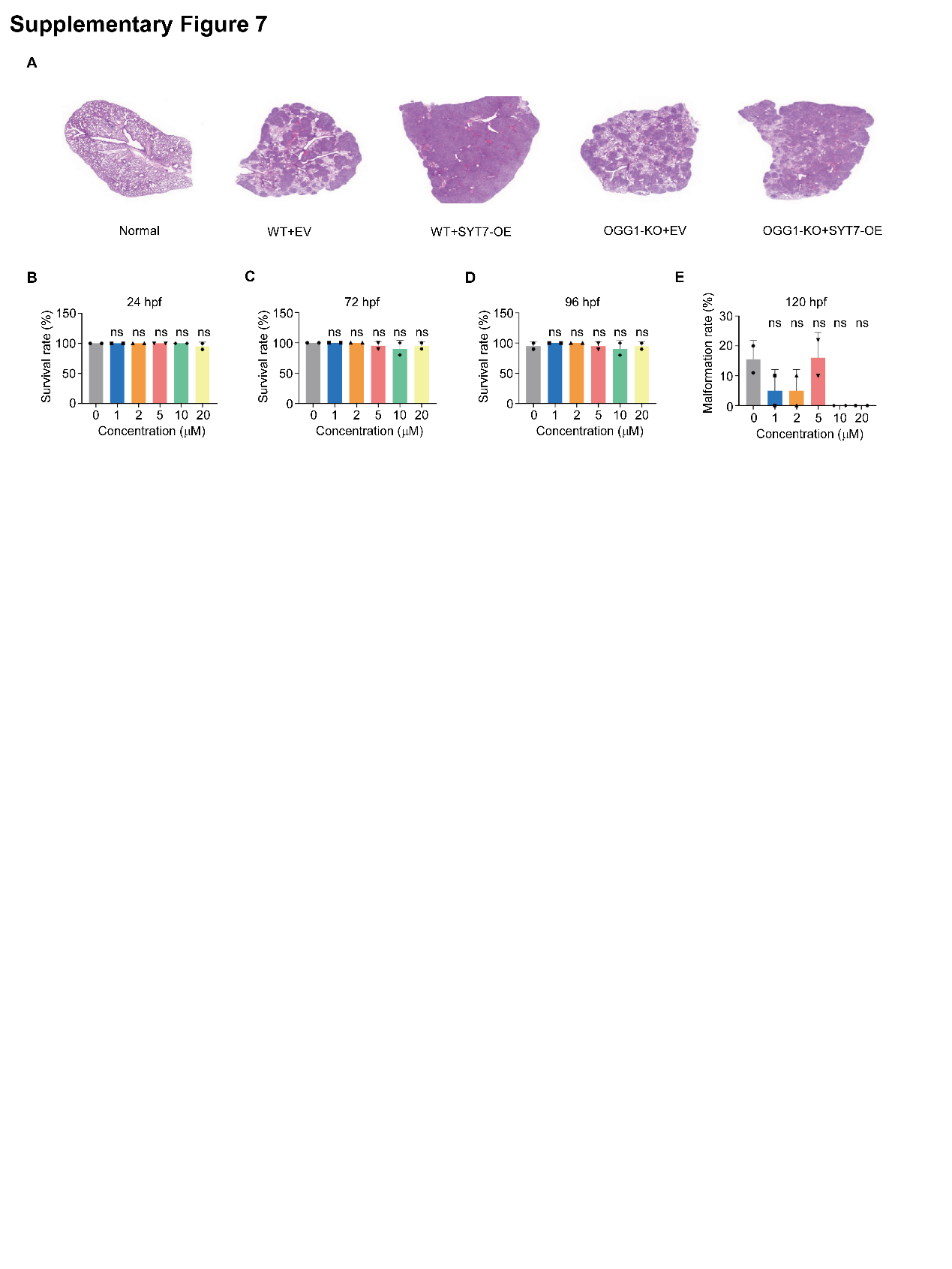


**Figure S7 OGG1 deletion reduces lung cancer metastasis in vivo.** (**A**) Representative photographs of whole lung slice scan. (**B-D**) Zebrafish juvenile survival rate at 24 (**B**), 72 (**C**), 96 (**D**) hpf. (**E**) Malformation rate of zebrafish larvae at 96 hpf. Conventional one-way *ANOVA* test was performed considering the experimental group as the independent variable. ns stands for no significance.

**Table S1:** **The primer sequences.**

| **Name** | **Sequence (5'-3')** | **Application** |
| --- | --- | --- |
| SYT7-F | TCATCACCGTCAGCCTTAGC | q-PCR |
| SYT7-R | TCTTGTAGCGTTTGCCCAGTT | q-PCR |
| OGG1-F | CACACTGGAGTGGTGTACTAGC | q-PCR |
| OGG1-R | CCAGGGTAACATCTAGCTGGAA | q-PCR |
| β-actin-F | CATGTACGTTGCTATCCAGGC | q-PCR |
| β-actin-R | CTCCTTAATGTCACGCACGAT | q-PCR |
| SYT7-CHIP-F | CGTGCTCCGCGGGCGGGCGGGA | CHIP |
| SYT7-CHIP-R | CTGCTCCGCCGCCGCCGCTGG | CHIP |
| G-SYT7-F/ Biotin-G-oligo-F | CGGGCGGGCGGGAGGGCTGGCGGGCGGCCCCC | EMSA/ Pull-down |
| G-SYT7-R-FAM/ 8-oxoG-SYT7-R-FAM | FAM-GGGGGCCGCCCGCCAGCCCTCCCGCCCGCCCG | EMSA |
| 8-oxoG-SYT7-F/ Cold probe-F/ Biotin-8-oxoG-oligo-F | CGGGCGGGCGGoGAGGGCTGGCGGGCGGCCCCC | EMSA/ Pull-down |
| Cold probe-R | GGGGGCCGCCCGCCAGCCCTCCCGCCCGCCCG | EMSA |
| Biotin-8-oxoG-oligo-R/ Biotin-G-oligo-R | Biotin- GGGGGCCGCCCGCCAGCCCTCCCGCCCGCCCG | Pull-down |
